# Biochemical impact of p300-mediated acetylation of replication protein A: Implications for DNA metabolic pathway choice

**DOI:** 10.1016/j.jbc.2025.110250

**Published:** 2025-05-17

**Authors:** Onyekachi Ononye, Sneha Surendran, Tripthi Battapadi, Pamela VanderVere-Carozza, Olivia K. Howald, Athena Kantartzis-Petrides, Matthew R. Jordan, Diana Ainembabazi, Marc S. Wold, John J. Turchi, Lata Balakrishnan

**Affiliations:** 1 Department of Biology, School of Science, Indiana University Indianapolis, Indianapolis, Indiana, USA; 2 Department of Medicine, Indiana University School of Medicine, Indianapolis, Indiana, USA; 3 Department of Pathology, Harvard Medical School, Boston, Massachusetts, USA; 4 Department of Biochemistry and Molecular Biology, Carver College of Medicine, University of Iowa, Iowa City, Iowa, USA

**Keywords:** replication Protein A (RPA), p300, lysine acetylation, single-strand DNA binding, G1/s phase, UV-induced damage

## Abstract

Replication Protein A (RPA), a single-stranded DNA (ssDNA) binding protein, is vital for various aspects of genome maintenance such as replication, recombination, repair, and cell cycle checkpoint activation. Binding of RPA to ssDNA protects it from degradation by cellular nucleases, prevents secondary structure formation, and suppresses illegitimate recombination. In our current study, we identified the acetyltransferase p300 to be capable of acetylating the 70 kDa subunit of RPA *in vitro* and within cells. The acetylation status of RPA changes throughout the cell cycle, increasing during the S and G2/M phases, and after UV-induced damage. Furthermore, we were able to specifically identify RPA directly associated with the replication fork during the S phase and UV damage to be acetylated. Based on these observations, we evaluated the impact of lysine acetylation on the biochemical properties of RPA. Investigation of binding properties of RPA revealed that acetylation of RPA increased its binding affinity to ssDNA compared to unmodified RPA. The improvement in binding efficiency was a function of DNA length with the greatest increases observed on shorter length ssDNA oligomers. Enzymatic assays further revealed that upon acetylation RPA governs the switch between the short and long flap pathway for Okazaki fragment processing. Our findings demonstrate that p300-dependent, site-specific acetylation enhances RPA’s DNA binding properties, potentially regulating its function during various DNA transactions.

Replication protein A (RPA) is a highly conserved heterotrimeric protein in eukaryotes and is involved in various aspects of DNA metabolism, such as DNA replication, repair, and recombination (1). Present at relatively high concentrations within human cells of ∼1 μM (2), it functions as the major single-strand DNA (ssDNA) binding protein (3). During various DNA transactions, high-affinity binding of RPA to ssDNA stabilizes the DNA and protects it from degradation from cellular nucleases (4). Stabilizing ssDNA structure by RPA also prevents the formation of stable secondary structures that could impede DNA transactions (5). Additionally, RPA serves as a platform for the assembly of a multitude of replication and repair-associated proteins during various biological events. RPA in eukaryotes is comprised of three subunits: RPA1, RPA2, and RPA3, with molecular weights of 70 kDa, 32 kDa, and 14 kDa, respectively (6). All three subunits are necessary to form a stable and functional RPA complex.

RPA1 has four oligosaccharide/oligonucleotide binding (OB) domains termed DNA binding domains (DBDs) A, B, C, and F, while RPA2 and RPA3 have one OB domain each–DBD-D and DBD-E, respectively (4, 7). Different RPA DBDs are activated depending on the length of the ssDNA bound to it (8). It was previously suggested that RPA shows a sequential mode of interaction with ssDNA; (i) low-affinity binding to ∼8 nt ssDNA where DBD-A and DBD-B of RPA1 would interact (9), (ii) medium-affinity binding to ∼18 to 20 nt ssDNA with DBD-A, -B and -C of RPA1 interacting, and (iii) high-affinity binding to ∼28-30 nt ssDNA which implicated all three DBDs of RPA1 (A, B, C) and additionally DBD-D of RPA2 (9). However, multiple recent studies have updated the modular binding model to propose a dynamic binding model for RPA to ssDNA, wherein the binding of RPA to the substrate is stable; however, it must be dynamically bound such that it easily hands-off the ssDNA substrate to its interacting protein partners (10). This model has been further improved in a study using hydrogen-deuterium exchange mass spectrometry (HDX-MS) to show dynamic binding by DBD-A and DBD-B and more stable binding by the TriC core made up of DBD-C, -D, and -E (11).

During DNA replication, unwinding the duplex DNA necessitates binding and protection by RPA on both the leading and the lagging strand ssDNA templates (12, 13, 14). On the leading strand, RPA inhibits priming by DNA polymerase alpha/primase (pol α); however, on the lagging strand, the priming function of pol α is stimulated in the presence of RPA (15). Additionally, RPA also influences lagging strand synthesis by stimulating the strand displacement activity of DNA polymerase δ (pol δ), which functions to create a 5′ flap structure (16). This structure is usually recognized, bound, and cleaved by flap endonuclease 1 (FEN1) (17). However, the creation of long 5′ flaps permits stable binding of RPA to displaced flaps, preventing FEN1 cleavage (18, 19). Processing of RPA-bound 5′ flaps requires the nuclease/helicase, Dna2, to displace RPA and cleave the flap to a length that is not optimal for RPA rebinding (20). FEN1 then cleaves the remainder of the flap, allowing for ligation and maturation of the Okazaki fragments. Thus, RPA functions as a governing switch that dictates the choice of flap processing during Okazaki fragment maturation (OFM) (18).

Furthermore, RPA plays an equally important role in DNA repair. RPA has been implicated in the base excision repair (BER) pathway as it physically interacts with Uracil DNA glycosylase (UNG) (21, 22, 23), and stimulates the long flap BER pathway (21). RPA ssDNA binding activity is also required for nucleotide excision repair (NER), where RPA binds to the undamaged strand of duplex DNA containing bulky DNA adducts (24, 25, 26). DNA double-strand break (DSB) repair can be accomplished *via* two main pathways: non-homologous end joining (NHEJ) and homologous recombination (HR) (27). RPA is one of the proteins that, when phosphorylated, works with phosphorylated Rad51 to find a homologous region to repair the damaged strands (28). There is also some evidence implicating other RPA post-translational modifications that could be involved in DNA repair *via* HR, but the exact details are unknown (29).

The ssDNA binding function of RPA and its interaction with protein partners are regulated within the cell using a variety of post-translational modifications (PTMs). The most extensively characterized modification is RPA phosphorylation, specifically on the N terminus of the RPA2 subunit. Differential phosphorylation of RPA2 occurs during various phases of the cell cycle, with phosphorylation of RPA2 linked to the G1 to S transition (30) and dephosphorylation linked to the mitotic phase (31). RPA2 is also phosphorylated on exposure to various DNA damaging agents such as hydroxyurea (HU) and UV irradiation, suggesting a role for phosphorylation in mitigating the damage response and NER (6, 32). Interestingly, lysine residues on RPA1 are also subject to multiple modifications, some of which may function as competing changes. For example, RPA1 is known to undergo acetylation, methylation, SUMOylation (33), ubiquitinylation (34), and crotonylation (35). SUMOylation of RPA1 has been demonstrated to aid in Rad51 recruitment to the location of damage to facilitate repair by homologous recombination (33). Another PTM observed on all three subunits of RPA is ubiquitination (34). Following DNA damage, RPA-ssDNA recruits Ataxia telangiectasia and Rad3-related protein (ATR), ATR interacting protein (ATRIP) kinase, and Pre-MRNA Processing Factor 19 (PRP19) complex to trigger phosphorylation and ubiquitination of RPA, which in turn activates ATR-ATRIP and the DNA damage response (DDR) (34, 36). As a part of the UV damage response, RPA was shown to undergo lysine acetylation (37) which altered the efficiency of NER (37). Characterization of RPA1 crotonylation in response to camptothecin (CPT)-induced DNA damage showed that the modification greatly enhanced RPA’s interaction with ssDNA (35). While lysine mono-methylation of RPA1 has been identified in proteomic analysis, there are no reports characterizing the impact of this modification on biological or cellular activity.

Our interest in the regulatory role of lysine acetylation on protein function arose from observing that many proteins involved in lagging strand maturation undergo this modification. Acetylation had contrasting effects on FEN1 and Dna2: it reduced FEN1’s nuclease activity (38) but enhanced Dna2’s cleavage function (39). Since RPA is known to govern the choice of the lagging strand maturation pathway, in our current work, we characterized the impact of lysine acetylation on the binding property of RPA. Contrary to previous reports, we found that in addition to PCAF and GCN5, RPA1 is also acetylated by the acetyltransferase p300 *in situ* and *in vitro* (36, 40). The main role of p300 is to act as a transcription coactivator that aids in chromatin remodeling, making it accessible for transcription (41, 42). Additionally, p300 has also been shown to acetylate many DNA replication and repair proteins, including FEN1 (39), Dna2 (39), WRN (43), DNA polymerase δ (pol δ) (44), and DNA polymerase β (45). Our work reveals additional lysine acetylation sites on RPA1 than previously reported. We also found the levels of RPA1 acetylation to increase in the S phase of the cell cycle, which is maintained through the G2/M phase. Similar to previous reports, we observed increased RPA acetylation on exposure to UV damage. Moreover, we showed a direct association of acetylated RPA1 with replicating and damaged forks. We further analyzed alterations in the dynamic binding of RPA on lysine acetylation and found that acetylation stimulates the binding activity of RPA to bind stably to shorter-length ssDNA. The acetylated form of RPA (Ac-RPA) was also slower in dissociating from the ssDNA than the unmodified form of RPA (Um-RPA) in the presence of a high excess of competing substrate. The melting and annealing properties of RPA are also influenced by lysine acetylation. Biochemical assays revealed Ac-RPA stimulated the formation of long flaps by stimulating Pol δ synthesis and strand displacement properties. Furthermore, Ac-RPA promoted long flap processing even on short DNA flaps by stably binding to it, thereby inhibiting FEN1 binding, and cleavage activities. Finally, we observed that RPA acetylation impacts the cleavage pattern of Dna2, ultimately affecting the maturation of OFs upon ligation. Our data shows that lysine acetylation of RPA is a key regulator of its function during various DNA transactions.

## Results

### *In vivo* and *in vitro* acetylation of RPA by acetyltransferase, p300

**Figure 1:**
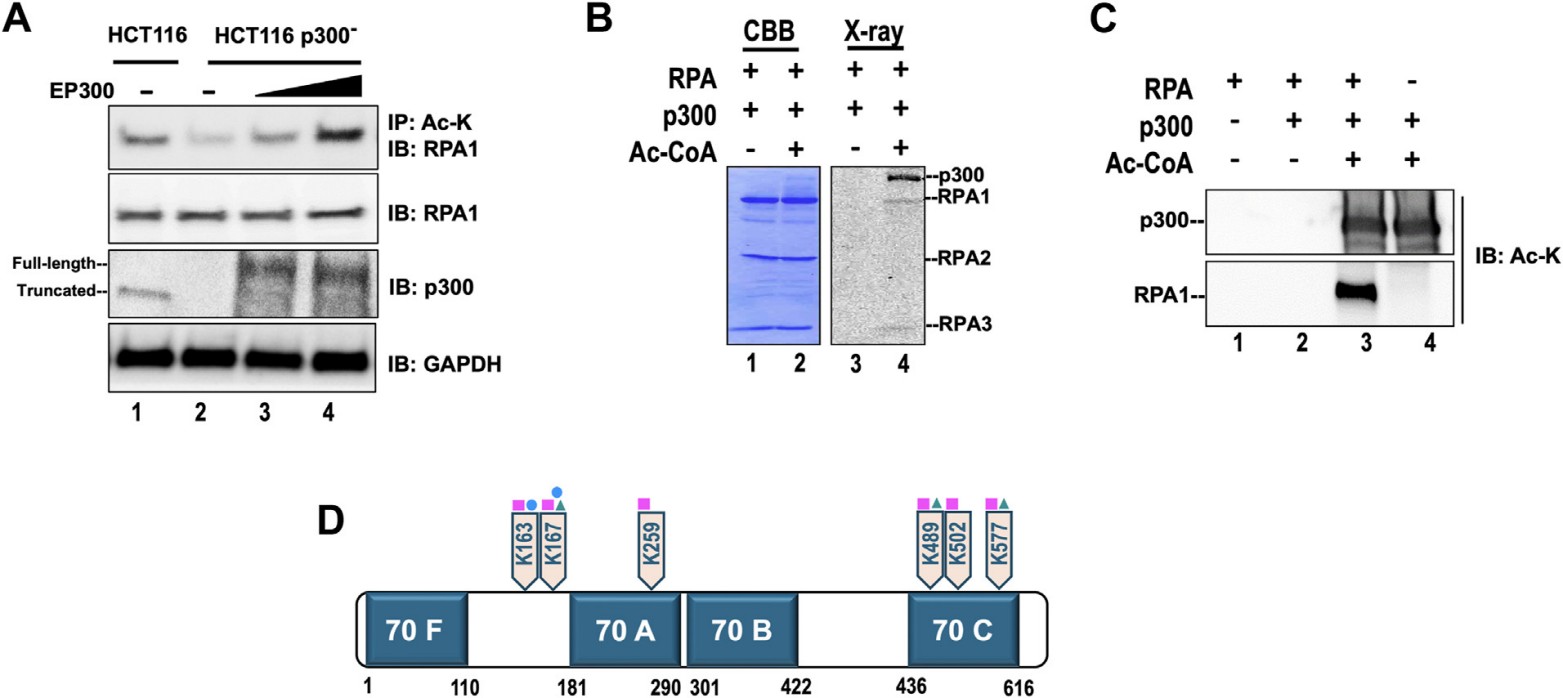
**Acetylation of RPA1 Subunit.***A*, IP-western blot analysis of RPA1 acetylation in wild-type HCT116, HCT116 p300^-^ and HCT116 p300^-^ cells rescued with EP300 expression. *B*, *in vitro* acetylated RPA (Ac-RPA) was subjected to SDS-PAGE analysis and stained using Coomassie brilliant blue (CBB). The same gel was subsequently analyzed by autoradiography (X-ray). *C*, *in vitro* acetylation of RPA and full-length p300 was visualized by Western blot analysis using an anti-acetyl lysine antibody. *D*, domains of Replication Protein A Subunit 1. Full length RPA was modified by *in vitro* acetylation using full-length p300 and subject to MS/MS mass spectrometry. Acetylated lysine residues on RPA1 and their positions are denoted. Competing modifications on the same lysine residue previously reported by proteomic studies are indicated, ubiquitination (square), sumoylation (triangle) mono-methylation (circle), and crotonylation (star).

A study analyzing the acetylation status of proteins in a whole cell extract using high-resolution mass spectrometry (MS) identified lysine (K) acetylation on residues K163, K167 and K259 of hRPA1 (37). A subsequent study using serial enrichments of different post-translational modifications found that in addition to RPA1 (K163 and K577), RPA3, the 14 kDa subunit, (K33, K39) was also acetylated (38). Other curated proteomic studies have determined K196, K267, K489, and K502 to be acetylated on RPA1 and noted on PhosphositePlus website (39). In studies using HEK293T cells, the RPA1 subunit was co-transfected with different acetyltransferases, and only GCN5 and PCAF was reported as capable of acetylating RPA1 (40, 41). Since numerous proteins in the DNA replication and repair pathway were modified by another lysine acetyltransferase (KAT), p300, and because proteomic analysis identified acetylation signatures on RPA1 that could potentially be linked to p300 related signatures, we tested the ability of p300 to acetylate RPA. To assess endogenous cellular RPA acetylation, in the presence and absence of p300, we used the colon cancer p300 wild-type (wt) cell line HCT116 and a p300 deficient cell line (HCT116^p300-^) derived by targeting the exon two of the P300 gene. Additionally, we also assessed acetylation of RPA in HCT116^p300-^ cell line, transfected with increasing concentrations (1 μg and 2.5 μg) of plasmid expressing EP300 cDNA (HCT116^p300-^ + EP300). We observed RPA1 to be acetylated in all three cell lines using immunoprecipitation followed by western blot analysis. Acetylation levels of RPA1 were measured by immunoprecipitating proteins using a pan-acetyl lysine, followed by immunoblotting with RPA1 antibody. Comparing the acetylation levels of RPA1, we observed ∼ 2-fold reduction in the acetylation levels of RPA1 in HCT116^p300-^ compared to wtHCT116 cells (Fig. 1*A*). Upon transfection with increasing concentrations of EP300 cDNA into the HCT116^p300-^ cells, we observed a corresponding increase in levels of acetylated RPA1 (Fig. 1*A*). This observation indicates that p300 is fully capable of acetylating endogenous RPA. Although RPA1 showed decreased level of acetylation in the p300-deficient cell line, compared to wild-type, we still observed low levels of RPA1 acetylation suggesting that in addition to p300, other redundant acetyltransferases are capable of acetylating RPA. Expression levels of the other KATs in both the wt and the p300 deleted cells are shown in Figure S1*A*. We repeated this experiment in HEK293T cells and observed a p300 associated dose-dependent increase in levels of endogenous RPA1 (Fig. S1*B*). We also assessed acetylation status of RPA2 and RPA3 from both HCT116 and HEK293 cells but did not detect any lysine acetylation on these subunits as assessed by western blot analysis (*data not shown*). *In vitro* acetylation of RPA1 by p300 was further confirmed using autoradiography (Fig. 1*B*) and western blot analysis (Fig. 1*C*). Unmodified RPA (Um-RPA) and acetylated RPA (Ac-RPA) – [modified using the catalytic domain of p300 and ^14^C-acetyl CoA] were subjected to separation by SDS-PAGE gel electrophoresis and stained using Coomassie Brilliant Blue (CBB). We detected all three subunits of RPA on the stained gel (lanes 1, 2, Fig. 1*B*). Autoradiography of the same gel revealed that in addition to the autoacetylation of the catalytic domain of p300, RPA1 and RPA3 (to a small extent) were also acetylated (lane 4, Fig. 1*B*). We also analyzed RPA acetylation by western blot analysis using a pan acetyl lysine antibody and found RPA1 to be robustly acetylated by p300 (lane 3, Fig. 1*C*) and the full-length p300 (lanes 3, 4, Fig. 1*C*) to be autoacetylated. We did not detect the acetylation of either RPA2 or RPA3 by Western blotting (data not shown).

To study the impact of RPA acetylation on its biochemical properties, we used the full-length acetyltransferase, p300, to *in vitro* modify full length RPA containing all three subunits. Sites of acetylation on the p300 modified RPA were determined using tandem mass spectrometry (MS/MS) on tryptic peptides. All peptide masses matched theoretical masses for tryptic peptides for human RPA. Spectra for acetylated peptides showed a mass change of +42 Da indicating addition of an acetyl group (Fig. S1*C*). Lysine sites that were previously identified to be acetylated on RPA1 in the proteomic analysis of whole cell extracts were also identified in our mass spectrometry analysis (K163, K167, K259, K489, K502, and K577). We were unable to identify any lysine residues on RPA2 or RPA3 that were *in vitro* acetylated by p300. However, this does not imply that these sites are not modified *in vitro*, as the absence of peptides containing these acetylated sites could be due to the poor ionization of the acetylated peptide, or the mass of the peptide being out of range for the set experimental values. Stoichiometric values for the extent of acetylation were not determined in these experiments. Lysine residues K167, K167, K259, and K577 were previously reported to also undergo another form of acylation, in the form of crotonylation (35). Additionally, proteomic studies (39) have also identified lysine residues K163, K167, K259, K489, K502, and K577 on RPA1 to be potential targets for other forms of post-translational modifications such as ubiquitination (square), sumoylation (triangle), and mono-methylation (circle) (Fig. 1*D*), suggesting that within the cell there could be competing or combinatorial PTM on the protein.

### RPA acetylation varies throughout the cell cycle and is highest in S-phase

Cellular events dictate the PTM status of proteins, altering protein properties (function, stability, and localization) and thereby enhance the repertoire of the cellular proteome. Since RPA is vital to DNA replication stability and fidelity, we first determined if cell cycle impacts the acetylation status of RPA. In order to directly assess acetylation of RPA, we developed an antibody recognizing acetylated K163 of RPA1 (RPA1_Ac-K163_). This antibody eliminated the need to immunoprecipitate RPA followed by immunoblotting using a pan-acetyl antibody. Specificity of the antibody to detect the K163 acetylated residue of RPA1 was confirmed using ELISA (Fig. S2*A*). Further, recombinant proteins (wild-type RPA, RPA1-K163R and RPA1-K167R) were *in vitro* acetylated using p300 and the specificity of the RPA1_Ac-K163_ antibody further confirmed using western blot analysis (Fig. S2*B*). However, while anti- RPA1_Ac-K163_ only detects a 70 kDa band on *in vitro* modified samples, it also detects ∼ a 55 kDa band in cellular extracts, which corresponds to a proteolytic product (Fig. S2*C*). This proteolytic product was shown to be stabilized on binding to ssDNA (42).

**Figure 2:**
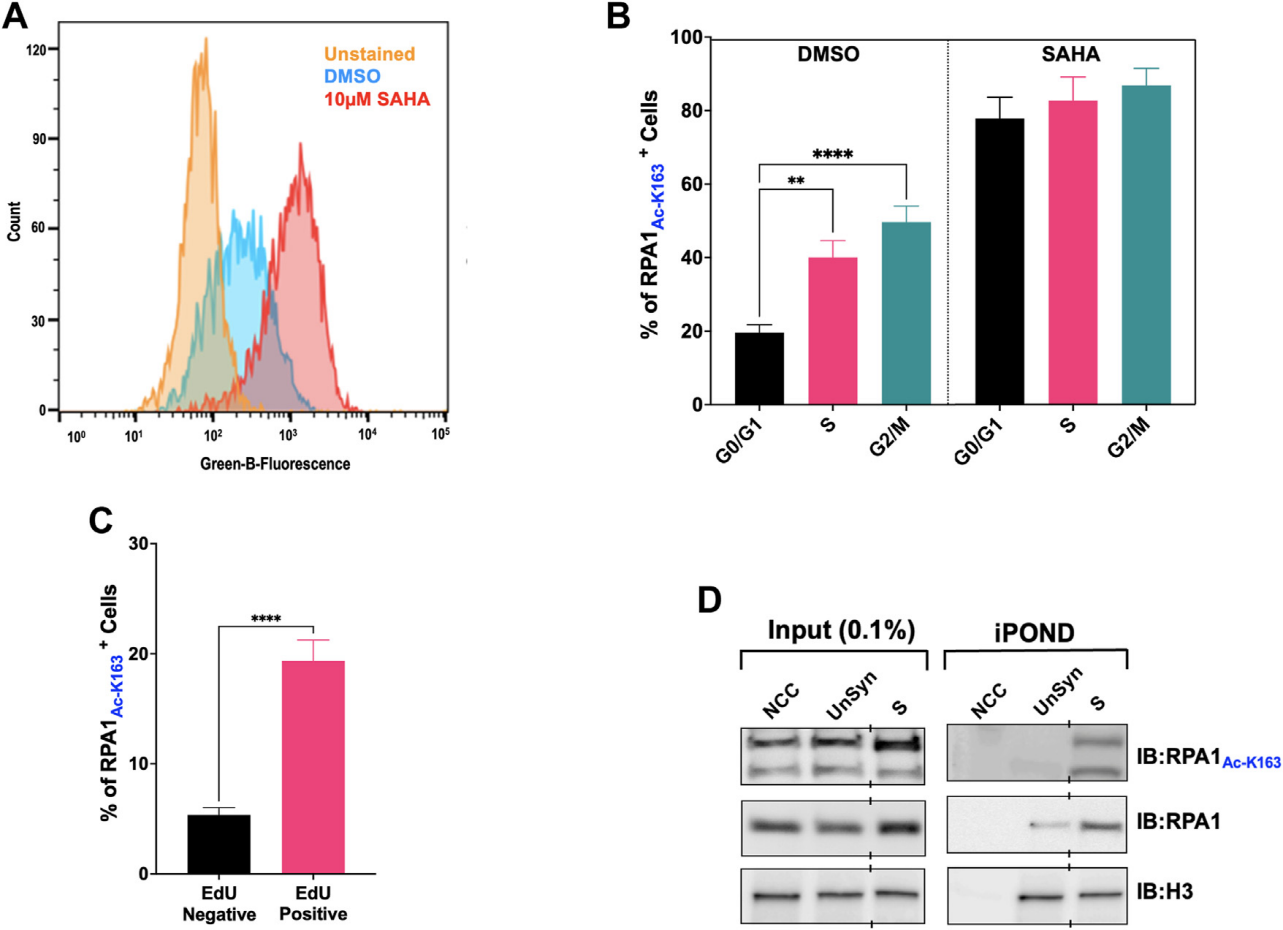
**Acetylation of RPA1 is cell cycle dependent.***A*, HEK293T cells were treated for 2 h with DMSO control or 10 μM SAHA. Staining of RPA1Ac-K163 as detected by flow cytometry is shown on a log scale on the X-axis vs count on the Y-axis. Plots for unstained, DMSO, and SAHA-treated cells were overlayed on the same graph using FlowJo software. *B*, graphical representation of the change in levels of acetylated RPA1 (RPA1_Ac-K163_) during different cell cycle phases in HEK293T cells. Statistical significance was assessed by one-way ANOVA ∗∗ *p*-value < 0.05, ∗∗∗∗ *p*-value < 0.001. *C*, graphical representation of RPA1 acetylation in EdU negative and positive HEK293T cells. Statistical significance was assessed by unpaired *t* test, ∗∗∗∗ *p*-value < 0.0001. *D*, either unsynchronized or S-phase synchronized HEK293 cells were subject to an iPOND assay. Lysates from the analysis were evaluated for the presence of acetylated RPA1 on the nascent replication strand.

Further characterization of the RPA1_Ac-K163_ antibody was obtained *via* indirect immunofluorescence. Results obtained revealed nuclear localization, as expected. In addition, fluorescence was increased upon treatment with a deacetylase inhibitor, suberoylanilide hydroxamic acid (SAHA) (Fig. 2*A*), showing the antibody was sensitive to changes in RPA1 acetylation levels. To assess RPA1 acetylation status as a function of cell cycle, exponentially growing asynchronous HEK293T cells were plated and treated with DMSO or 10 μM SAHA for 2 h. Cells were then processed for flow cytometric detection of acetylated RPA and total DNA content as described in “*Experimental Procedures*” (cell cycle profile shown in Fig. S2*D*). The data demonstrate a clear signal for Ac-RPA compared to the unstained cells and a significant increase in RPA acetylation upon treatment with SAHA, as expected (Fig. S2*E*). In addition, acetylated RPA is observed in cells in all phases of the cell cycle. Quantification revealed an increase in acetylation of RPA as cells progressed into S-phase and this level was maintained throughout G2 (Fig. 2*B*). Interestingly, when deacetylase activity was blocked, RPA showed similarly increased levels in all phases, suggesting that during the cell cycle, RPA1 acetylation is dynamically regulated during the cell cycle. To more accurately assess acetylation in S-phase, cells were pulse labeled with EdU for 30 min after SAHA or vehicle treatment. The data demonstrate that RPA is highest in EdU-positive S-phase cells (Fig. 2*C*). Staining profiles are shown in Figure S2*F*. These data demonstrate that in unperturbed cell cycle progression, RPA acetylation increases as cells progress from G1 to S-phase, is largely maintained through G2, and is reset in M to give the lower level observed in G1.

To assess if RPA is acetylated while functioning at the replication fork, we performed an isolation of proteins on nascent DNA (iPOND) assay. In the unsynchronized cell population (lane 5, Fig. 2*C*), we detected a faint product corresponding to acetylated RPA. However, this product was significantly enriched when cells were synchronized in the S phase using a double thymidine block (lane 6, Fig. 2*C*). This observation confirms acetylated RPA is present directly at the replication fork.

### RPA is acetylated in response to DNA damage repair

**Figure 3:**
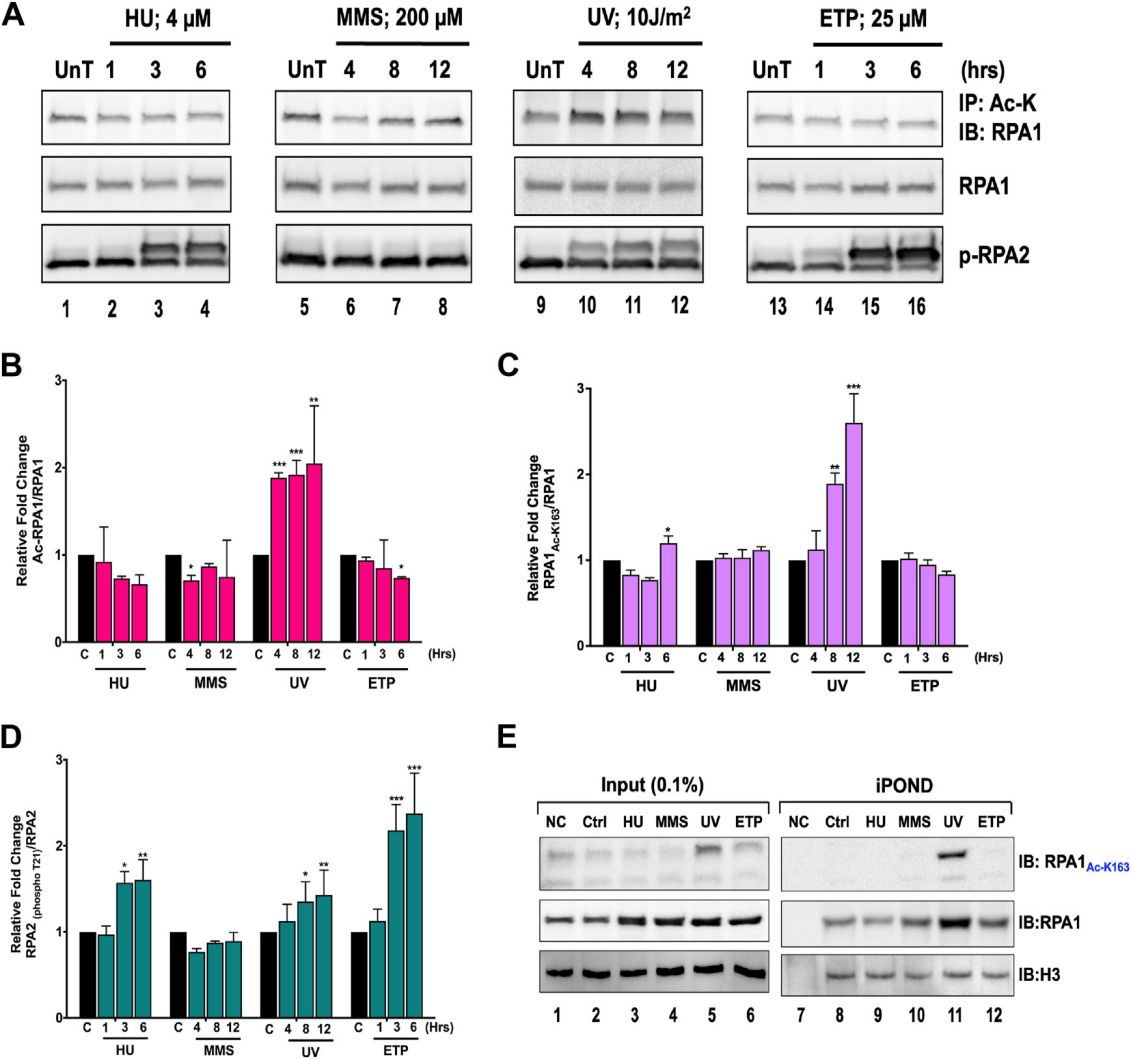
**Acetylation is Triggered on UV Damage to the Cells.***A*, HEK293T cells were treated with different DNA-damaging agents as described in the Materials and Methods. Cell lysates from the different treatments were immunoprecipitated using a pan acetyl-lysine antibody and immunoblotted using RPA1 specific antibody. Graphical representation of (*B*) change in levels of acetylated RPA1, (*C*) cell lysates treated with different DNA-damaging agents were analysed by western blot using the RPA1_Ac-K163_ antibody, with corresponding changes in RPA1K163 acetylation levels represented graphically. *D*, phospho-RPA2 on treatment with different DNA-damaging agents in HEK293T cells, *E*, HEK293 cells treated with different DNA-damaging agents (HU and ETP – 6 h, MMS and UV – 12 h), were subject to an iPOND assay. Lysates from the analysis were evaluated for the presence of acetylated RPA1 on the nascent replication strand. Figures [*A*] and [*E*] are representative gels, and the error bars in [*B*–*D*] are for the average of three independent experiments. Data is represented as Mean + SEM. Statistical significance was assessed by two-way ANOVA, ∗ *p*-value < 0.05, ∗∗ *p*-value < 0.005, ∗∗∗ *p*-value < 0.001.

Next, we assessed if DNA damage regulates the acetylation of RPA, since both Dna2 and FEN1 show increased acetylation on UV damage (43). HEK293T cells were exposed to various DNA-damaging agents, including hydroxyurea (HU), methyl methanesulfonate (MMS), ultraviolet radiation (UV), and etoposide (ETP), and the change in acetylation pattern of RPA1 was analyzed. DNA damage was confirmed by the presence of markers such as phospho Chk1 (p-Chk1), phospho Chk2 (p-Chk2), phospho p53 (p-p53), and phospho H2AX (p-H2AX) (Fig. S3*A*). Similar to both Dna2 and FEN1, RPA1 also showed an increase in acetylation on exposure to UV radiation and did not display a detectable increase in acetylation in response to other forms of damaging agents (Fig. 3*A*). RPA1 levels were normalized to one and fold increase in levels of acetylated RPA1 levels calculated and plotted in Figure 3*B*. To circumvent any bias of co-immunoprecipitation, we also performed western blot analysis of cell lysates treated with different DNA-damaging agents and probed with the RPA1_AC-K163_ antibody. Consistent with the findings in Figure 3*B*, UV-induced DNA damage led to increased RPA1K163 lysine acetylation (Figs. 3*C* and S3*B*). Alterations in the phosphorylation of RPA2 serve as a control for known changes in response to DNA damaging agents (Fig. 3*D*). Cells are known to undergo global hyperacetylation in response to either MMS (44) or UV damage (45). We were interested in determining if the increase in cellular pools of acetylated RPA1 could be correlated to the RPA that is directly associated with the damaged fork. Using the iPOND assay, we probed for the acetylation status of RPA1 directly associated with the damage fork using anti-RPA1_Ac-K163_ and found that acetylated RPA1 correlated directly with repair of UV damaged forks (Fig. 3*E*). Overall, our results suggest that similar to checkpoint kinases that are activated in DNA damage response, RPA1 acetylation could specifically be involved in mediating the UV-induced damage response.

### Acetylation of RPA increases its ssDNA binding affinity

**Figure 4:**
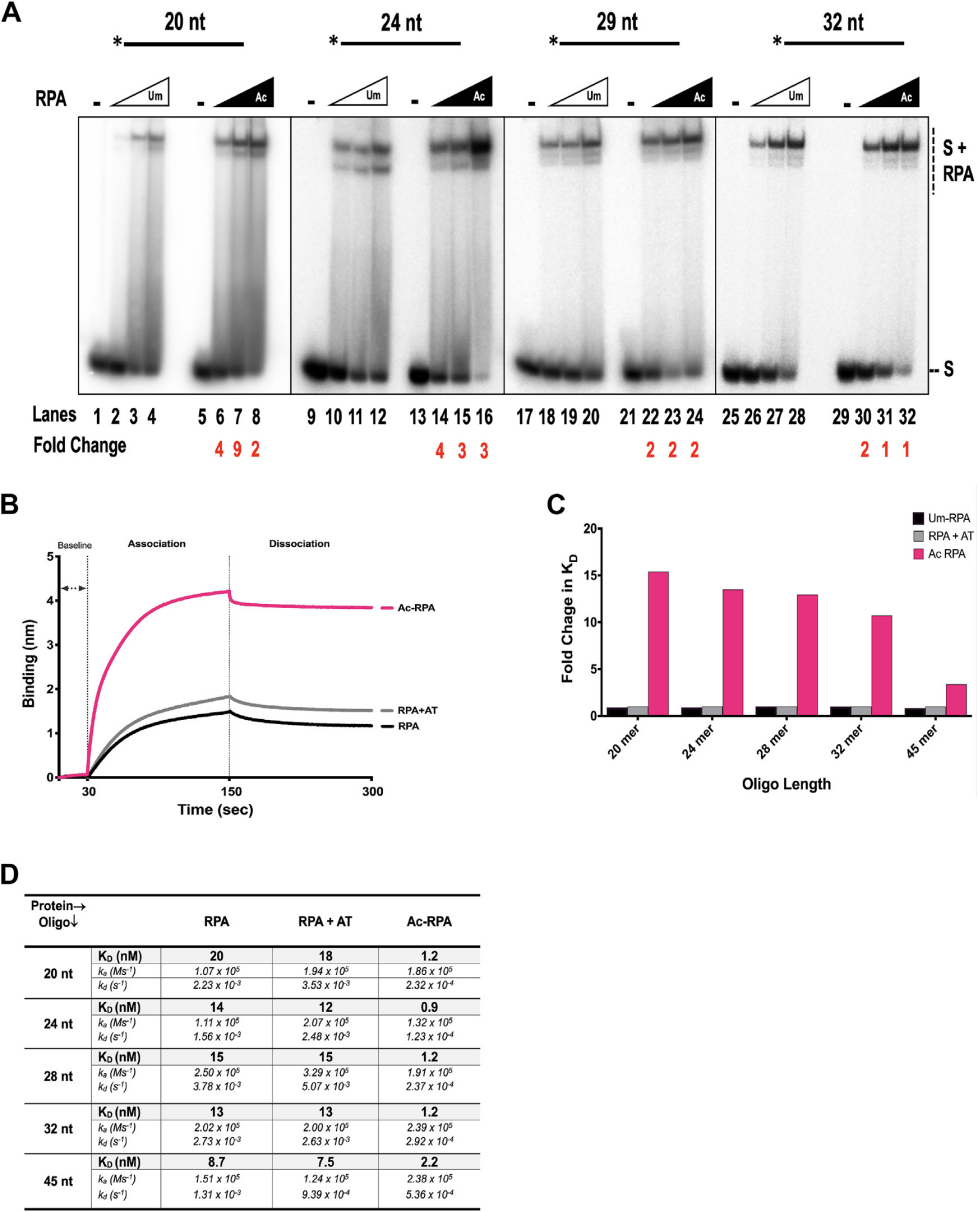
**Characterizing the binding property of acetylated RPA.***A*, increased Binding Efficiency of Ac-RPA. The binding efficiency of Um-RPA and Ac-RPA was studied using EMSA. Five nanomolar substrates of varying lengths (20, 24, 28, and 32 nt) were incubated with increasing concentrations (5, 10, and 25 nM) of Um-RPA or Ac-RPA, and the reactions were incubated for 10 min at 37 °C, and subsequently separated on a 6% polyacrylamide gel. The labeled substrate is depicted above the gel with the asterisk indicating 5′ of the ^32^P label. The substrate alone and the complexes containing RPA-bound substrate are indicated beside the gel at the *right*. The fold change in the binding of Ac-RPA compared to Um-RPA is denoted below the lane numbers. *B*, sensorgram obtained using streptavidin biosensor coated with 10 nM 28 nt biotinylated oligonucleotide incubated with 160 nM Um-RPA, Um-RPA + AT, and Ac-RPA. The coating of the biosensor and the association and dissociation curves are shown in the sensorgram. *C*, fold change in binding affinity of Um-RPA + AT and Ac-RPA compared to Um-RPA was calculated. The inverse fold change was then plotted graphically to show the stimulation in binding efficiencies of the Ac-RPA compared to Um-RPA and RPA + AT. *D*, data from the sensorgrams were fit globally to a 1:1 binding model to yield equilibrium dissociation constant (K_D_), association constant (ka), and dissociation constant (kd). Values for K_D_ measurements for different length oligos are listed in the table.

Binding of RPA to ssDNA is initiated by weak dynamic interactions at lengths of ∼ 8 to 10 nt involving the A and B domains, while high-affinity binding of RPA requires ssDNA to be ≥28 nucleotides. We tested the binding efficiencies of unmodified and acetylated RPA on different length oligomers (22, 24, 28 and 32 nt) using electromobility gel shift assays (EMSAs). Based on the length of the ssDNA and known binding properties of RPA, we expected weaker binding to the 22 and 24 nt oligomers compared to high affinity binding to the 28 and 32 nt oligomers. To measure binding, we titrated varying concentrations of either Um-RPA or Ac-RPA (5, 10, 25 nM) in the presence of ssDNA (5 nM) and incubated at 37 °C for 10 min. Following incubation, the reactions were loaded onto a 6% native gel, electrophoresed, and subsequently analyzed. Binding results indicated that RPA (unmodified and acetylated forms) showed binding to all four different length oligomers. Interestingly, irrespective of the length of the oligomer, the Ac-RPA showed higher binding efficiency compared to Um-RPA. However, it is important to note that the fold stimulation in binding of the acetylated form compared to the unmodified form correlated with the lengths of the oligomers. The 22 nt oligomer showed the highest fold stimulation in binding by Ac-RPA (compare lanes 6–8 to lanes 14–16, 22–24, 30–32, Fig. 4*A*). The smeared pattern of binding to the 22 and 24 nt oligomer is consistent with dynamic binding property of RPA to shorter oligonucleotides. Incubation of Um-RPA and p300 in the absence of acetyl coenzyme A showed that it bound similar to Um-RPA. The acetyltransferase, p300 was unable to bind to the substrate, suggesting that the observed shift was only due to RPA’s interaction with the substrate (Fig. S4). The control experiments show that the increased binding efficiency of Ac-RPA was due to lysine modification on the protein and not due to stabilizing interactions with the acetyltransferase.

To further characterize RPA-ssDNA interactions in real time, we used the label-free biolayer interferometry (BLI) technology to measure protein association and dissociation. Streptavidin biosensors were coated with 10 nM of different length biotinylated oligomers (20, 24, 28, 32 and 45 nt) for a period of 100 s and allowed to associate with specific concentrations of either unmodified RPA (Um-RPA), RPA incubated with acetyltransferase (AT), p300 (Um-RPA + AT), or acetylated RPA (Ac-RPA) for a period of 300 s and then moved to a buffer wherein dissociation was measured for a period of 300 s. The resulting sensorgram allowed measurement of association and dissociation rate constants (*k*_*a*_ and *k*_*d*_) and the equilibrium binding constant (K_D_). An example of the sensorgram showing the association and dissociation of 100 nM protein (Um-RPA, Um-RPA + AT, Ac-RPA) with a 28 nt oligomer is shown in Figure 4*B*. Measurements for binding of different concentrations of RPA with different length oligonucleotides were calculated and *k*_*a*_, *k*_*d*_, and K_D_ values determined (Fig. 4*C*). Measured binding constants of the unmodified form of RPA agreed with previously reported steady state measurements (46, 47, 48). For every tested length of oligonucleotide, we found that Ac-RPA had significantly lower K_D_ compared to the Um-RPA or the Um-RPA + p300 (in the absence of acetyl coenzyme A). While the *k*_on_ of both Um-RPA and Ac-RPA were fairly similar, the off-rate of the Ac-RPA was ∼ 10-fold slower by the Ac-RPA compared to the Um-RPA (Fig. 4*D*). Calculation of the fold change in K_D_ revealed that similar to the EMSA results, fold change in binding constant was the highest for the shortest length oligonucleotide (20 nt) and the lowest for the longest length oligonucleotide (45 nt) measured (Fig. 4*D*). The lower stimulation of binding for the longer length oligonucleotide was expected since RPA is capable of binding to oligonucleotides of this length with high affinity without the need for additional stimulation by acetylation.

To determine if there was a correlation between increased binding and the number of acetylation sites in the protein’s DBD, we tested mutants of RPA1 subunit containing varying number of DBDs and acetylation sites. The DBD-F mutant contained only DBD-F domain and the linker region with two acetylation sites (K163 and K167); the FAB mutant contained DBD-F, DBD-A and DBD-B with five acetylation sites (K163, K167, K259, K331 and K379); the A1/A2 mutant contained two DBD-A domains fused together and two acetylation sites (K259, K331) and ΔF-RPA mutant contained all DBDs except DBD-F as well as seven acetylation sites (K259, K331, K379, K443, K489, K502, and K577) (Fig. S5). *In vitro* acetylation of the RPA1 mutants was confirmed both by autoradiography and by tandem mass spectrometry (*data not shown*). Um- and Ac-RPA1 mutants were incubated with a 30 nt TAMARA-labeled ssDNA and their binding affinities were analyzed by EMSA. Both the unmodified and acetylated forms of DBD-F mutant did not bind to the substrate. This was an expected result, since the DBD-F does not contribute to ssDNA binding. However, this also confirms that acetylation on sites K163 and K167 do not cause an observable change in the binding property of DBD-F. Additionally, this result further shows that p300 does not complex with DNA to create a gel shift. Acetylation of all the other mutants (FAB, A1/A2, and ΔF-RPA) showed increased DNA binding compared to their corresponding unmodified forms (Fig. S5). Our results suggest that the acetylation of one or more lysine residues in RPA1 results in increased ssDNA-binding efficiency, irrespective of the location of the acetylation sites with respect to the DBDs.

### Acetylated RPA binds more tightly to its substrate compared to the unmodified form

**Figure 5:**
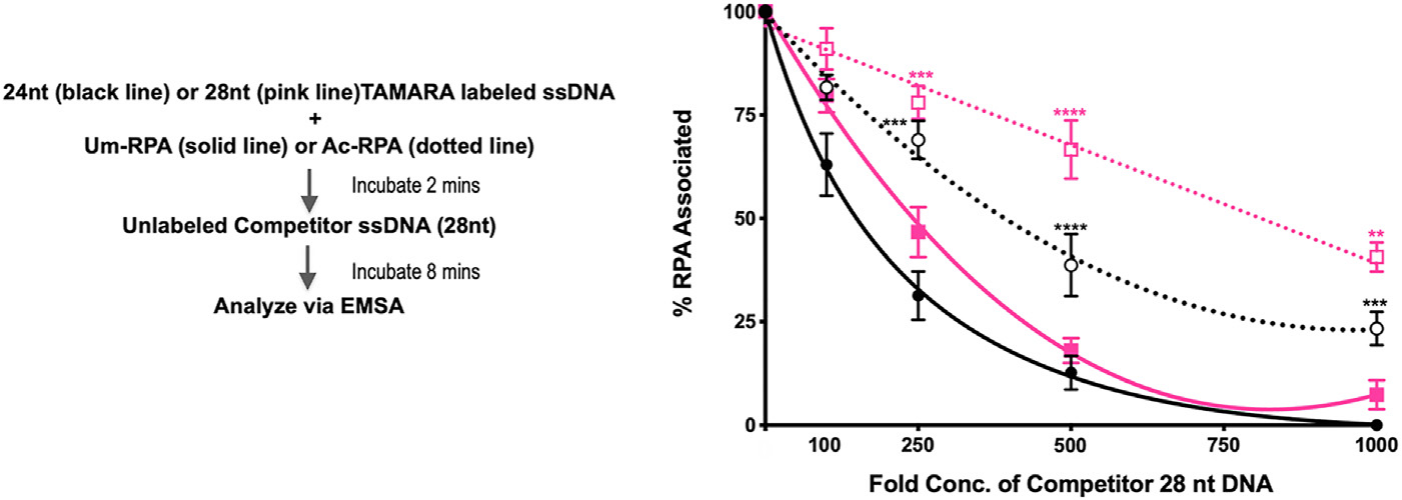
**Assaying the binding efficiency of acetylated RPA in the presence of competing ssDNA.** One hundred nanomolar Um-RPA (*solid line*) or Ac-RPA (*dotted line*) was pre-incubated with either a 24 nt (*black line*, round bullet) or 50 nM of Um-RPA or Ac-RPA was pre-incubated with 28 nt (*pink line*, square bullet) TAMARA-labeled ssDNA substrate for 2 min at 37 °C. To this reaction, 100-, 250-, 500-, and 1000-fold excesses of cold competitor 28 nt ssDNA were added, and the mixture was further incubated for 8 min at 37 °C and then separated on a 6% polyacrylamide gel. The percentage of protein dissociated from the substrate was calculated and graphically plotted to reveal the binding efficiency of Um-RPA and Ac-RPA in the presence of an excess competitor ssDNA. Data is represented as Mean + SEM. Significant two-way ANOVA effects denoted by ∗∗*p* < 0.05, ∗∗∗*p* < 0.001, ∗∗∗∗*p* < 0.0001.

**Figure 6:**
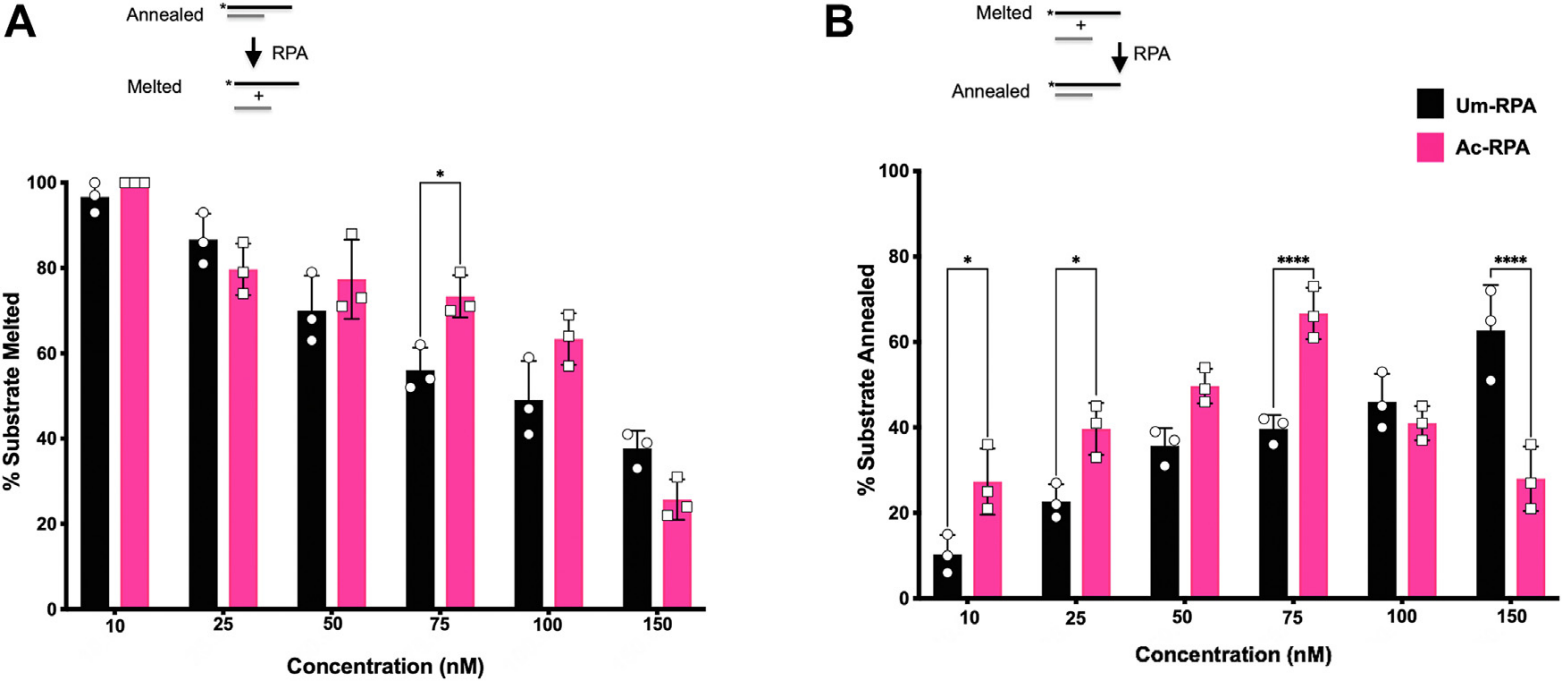
**Assessment of the annealing and melting properties of acetylated RPA.***A*, annealed duplex oligomers (37 + 57 nt) were incubated with varying concentrations (10, 25, 50, 75, 100, 150) of either Um-RPA or Ac-RPA for 30 min at 37 °C and separated on a 6% polyacrylamide gel to assess the melting properties of RPA. *B*, a 37 nt oligomer complementary to a 57 nt template was incubated in the presence of varying concentrations (10, 25, 50, 75, 100, 150) of either Um-RPA or Ac-RPA for 30 min at 37 °C and separated on a 6% polyacrylamide gel to assess the annealing properties of RPA. Annealing was normalized as a control that did not contain any RPA in the reaction. The data for annealing and melting reactions are graphically represented as mean + SEM (the average of three independent experiments). Significant two-way ANOVA effects denoted ∗ *p* < 0.05, ∗∗∗∗*p* < 0.0001.

It has been previously shown that ssDNA-bound RPA rapidly exchanges in the presence of free RPA (10); however, it can remain stably bound to the ssDNA for many hours (49). Given that many biological pathways are dependent on the assembly of RPA on ssDNA and the subsequent hand-off to its interacting protein partners, we were interested in comparing the dissociation of unmodified and acetylated RPA in the presence of a competitor ssDNA substrate. For the competition assays, we chose two ssDNA substrates, a 24 nt and 28 nt substrate. Since only substrates longer than ∼ 28 nt are bound efficiently by RPA, we expected weak binding on a 24 nt ssDNA and tighter binding to 28 nt ssDNA (50). We used higher concentrations of RPA (unmodified and acetylated) to prebind the 24 nt ssDNA substrate compared to the 28 nt ssDNA substrate, to ensure 100% binding of RPA on the shorter length substrate. We prebound either Um-RPA or Ac-RPA to a TAMARA-labeled 24 nt or a 28 nt ssDNA and allowed it to incubate for 2 min. We then introduced different fold excess (100, 250, 500, and 1000-fold) of a competitor substrate (unlabeled 28 nt substrate) and allowed it to incubate with the reaction for 8 min. The reactions were then analyzed using EMSA, and the results are graphically represented in Figure 5. The 28 nt competitor unlabeled ssDNA was able to compete off Um-RPA (solid line) from both the 24 nt (black line) and the 28 nt (pink line) substrates at much lower concentrations compared to the Ac-RPA (dotted line). In the presence of a 500-fold excess competitor, nearly 80% of the bound Um-RPA had dissociated from both the 24 nt (black solid line, Fig. 5) and 28 nt substrate (pink solid line, Fig. 5). However, at the same concentration of the competitor, only ∼33% of Ac-RPA was displaced from the 28 nt (pink dotted line, Fig. 5) substrate and ∼61% from the 24 nt substrate (black dotted line, Fig. 6). Similarly, when all of the bound Um-RPA was displaced in the presence of 1000-fold competitor, 77% of Ac-RPA was displaced from the 24 nt substrate (black dotted line, Fig. 5) and 60% from the 28 nt substrate (pink dotted line, Fig. 5). These data suggest that the Ac-RPA bound more tightly to the substrate and requires a significantly higher amount of competing substrate to be dissociated from its already bound state. These data also correlate with our *k*_*d*_ measurements wherein we observed a slower k_off_ of Ac-RPA compared to Um-RPA (Fig. 4*D*).

As RPA acetylation enhances DNA binding, we next evaluated the propensity of Ac-RPA to be inhibited by small-molecule RPA inhibitors (RPAi) (51, 52) that inhibit RPA-DNA binding and exhibit anticancer activity. We utilized two different RPAi, one that is well established (TDRL-551 (51)) and a second, novel RPAi (DA1-73, manuscript in preparation). We performed RPAi titrations at equimolar Um-RPA and Ac-RPA concentrations where the vehicle controls are ∼17% and ∼77% bound for Um-RPA and Ac-RPA, respectively (Fig. S6, *A* and *C*, lanes 2 and 8). Interestingly, both TDRL-551 (Um-RPA IC50 = 4.46 ± 0.11 μM, Ac-RPA IC50 = 14.12 ± 3.45 μM) and DA1-73 (Um-RPA IC50 = 6.43 ± 0.29 μM, Ac-RPA IC50 = 9.87 ± 2.10 μM) exhibit more potent inhibition of Um-RPA as compared to Ac-RPA (Fig. S6, *B* and *D*). However, on equal binding of Um-RPA (by incubating at a higher concentration compared to Ac-RPA) and Ac-RPA, the inhibitor was able to equally displace both Um-RPA and Ac-RPA, suggesting that acetylation did not directly interfere with inhibitor activity (data not shown). Thus, similar to the competitor assay (Fig. 5), the inhibitor assays show that Ac-RPA binds more tightly to the substrate compared to the Um-RPA.

### Lysine acetylation alters strand melting and annealing properties of RPA

The inherent binding property of RPA affords its subsidiary functions to either melt or anneal duplex DNA. Since helix destabilization has been linked to RPA1 (9, 53, 54), we were interested in studying the impact of lysine acetylation on this activity of the protein. Alterations in annealing and melting were assessed using the same substrates. To characterize the influence of lysine acetylation on RPA annealing, varying concentrations of the protein (10, 25, 50, 75 and 100 nM) were incubated with two complementary ssDNA oligonucleotides at 37 °C for 10 min. Our results reveal that there isn’t a significant difference in the amount of annealed products formed in the presence of either Um-RPA or Ac-RPA (Fig. 6*A*).

Similarly, to study strand melting, we first annealed duplex DNA and incubated increasing concentrations (10, 25, 50, 75, and 100 nM) of either Um-RPA or Ac-RPA with the substrate at 37 °C for 10 min. Melted products were observed by loading the completed reactions to a 6% native gel and calculating the % of ssDNA in each reaction. While we observed an increase in the melting properties of Ac-RPA compared to the Um-RPA, at the highest concentration of RPA (150 nM), we observed that Um-RPA had improved melting activity compared to Ac-RPA (Fig. 6*B*). This is likely because the melting and annealing properties of RPA are not intrinsic to the protein itself; as a result, the effect of acetylation on these properties may depend on the local concentration of RPA under the reaction conditions.

### Synthesis and strand displacement by DNA polymerase delta (pol δ) is stimulated in the presence of acetylated RPA

**Figure 7:**
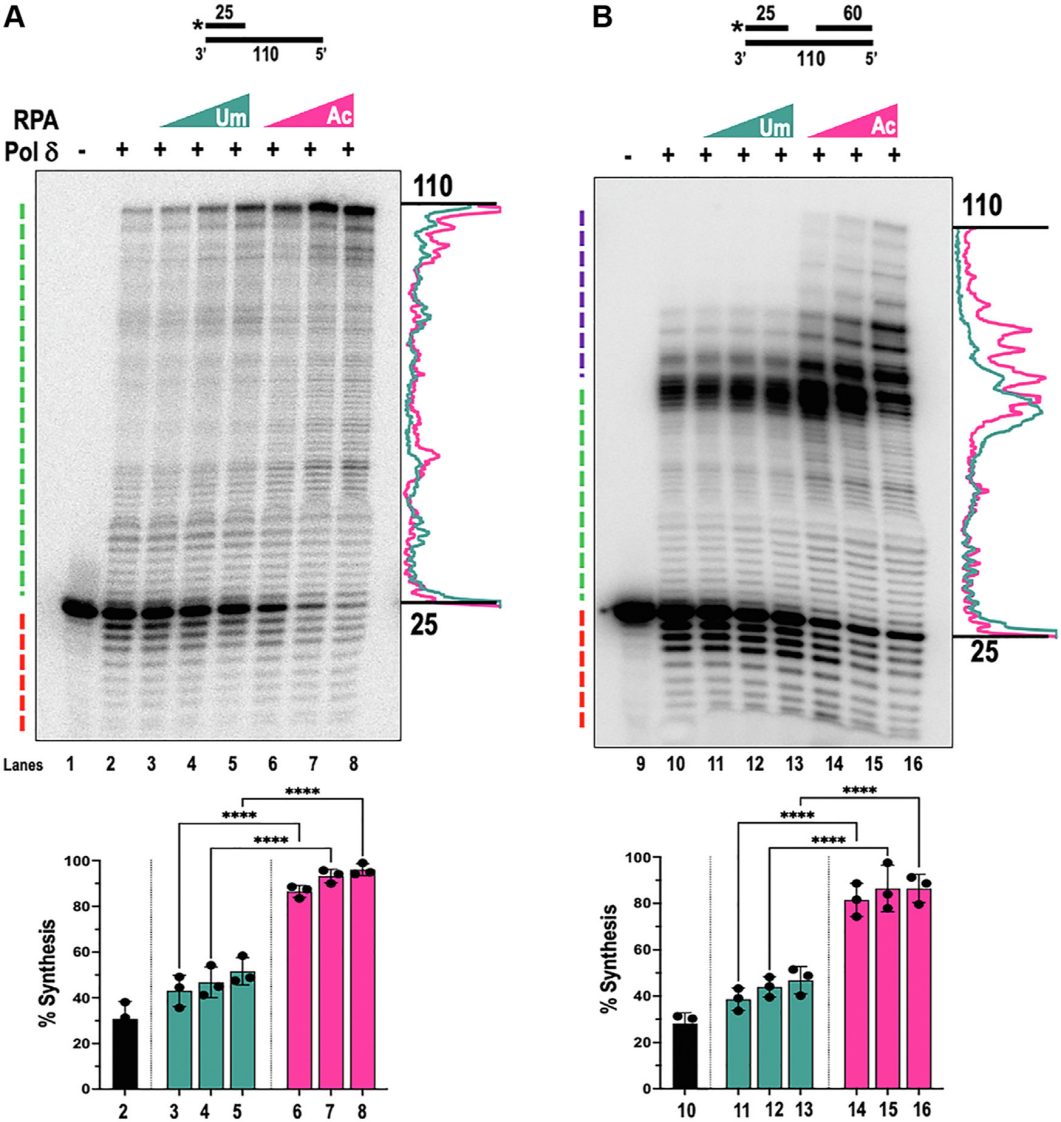
**Ac-RPA stimulates Pol δ synthesis, strand and displacement activities.** Pol δ (100 nM) synthesis was assessed on 5 nM of (*A*) synthesis (25U:110T, lanes 1–8) and (*B*) strand displacement (25U:110T:60D, lanes 9–16) substrates in the presence of increasing concentrations (50, 100, and 200 nM) of either Um-RPA or Ac-RPA. Synthesis and strand displacement profiles of Pol δ were assessed by separating the reactions on a 12% denaturing PAGE gel. Substrates are depicted above the gel, and with the asterisk denoting the position of the ^32^P radiolabel. Full-length synthesis (110 nt) and labeled substrate (25 nt) are denoted on the gel. Lines (on the *left* side of the gel) indicate exonuclease (*red*), synthesis (*green*), and strand displacement (*violet*) activities. Synthesis traces provided on the *right* side of the gel show the differences in Pol δ synthesis in the presence of Um-RPA and Ac-RPA. The percentage of overall synthesis was calculated and graphically plotted to align below each gel image. Data are represented as Mean + SEM. Significant two-way ANOVA effects denoted by ∗∗∗∗*p* < 0.0001.

Although the core intrinsic function of RPA is ssDNA binding, it has also been shown that RPA exhibits some strand-melting properties that aid in resolving secondary structures that limit the activity of some polymerases and helicases (9). Prior work in yeast and mammalian cells has revealed that the processivity of Pol δ is improved in the presence of certain accessory proteins, including PCNA and RPA (55). This increased processivity ensures that each Okazaki fragment is efficiently synthesized and that the preceding initiating primer is processed out of the genome, preventing the incorporation of ribonucleotides and mis-incorporated deoxyribonucleotides (56). RPA binding to the parent strand helps melt most secondary structures that could serve as a barrier to synthesis. Since Ac-RPA showed increased strand melting properties at lower concentrations (Fig. 5), we sought to explore the impact of RPA acetylation on the processivity of Pol δ as measured by the amount of synthesis, and strand displacement products formed from OFM-like substrates as shown in Figure 7.

To assess polymerase synthesis, we pre-bound varying concentrations (25, 50, and 100 nM) of Um-RPA or Ac-RPA to a synthesis substrate (Fig. 7*A*) and assayed for the synthesis activity in the presence of 150 nM of Pol δ. Our results revealed that Um-RPA was able to modestly stimulate the polymerase processivity and gap-filling activity at the tested concentrations. At the highest concentration of Um-RPA, we calculated ∼2-fold increase in the overall synthesis of Pol δ and a 2-fold increase in the formation of full-length 110 nt product (Fig. 7*A*, compare lane 5 to lane 2) In comparison we observed ∼3-fold increase in the overall synthesis in the presence of Ac-RPA and ∼ 6.5-fold increase in the formation of the full-length 110 nt product (Fig. 7*A*, compare full length-product in lane 2 to lane 8). Notably, full-length product formation with Ac-RPA was ∼ 3.2-fold higher than that observed with Um-RPA.

Using similar experimental conditions, the strand displacement activity of Pol δ was measured (Fig. 7*B*). On this substrate, as the upstream primer is labeled, we can measure gap-filling products as well as strand-displacement products generated by displacing the downstream primer. In the Pol δ alone lane (Fig. 7*B*, lane 10) or in the presence of the highest concentration of Um-RPA (Fig. 7*B*, lane 13), minimal strand displacement synthesis was observed. However, when overall synthesis was calculated in the presence of Um-RPA a minimal increase (∼1.5-fold) in synthesis past the 25 nt gap (Fig. 7*B*, lanes 11–13) was observed. However, in the presence of Ac-RPA, there was a higher stimulation to overall synthesis activity (∼3-fold) and a significant stimulation in the strand displacement activity (∼7.5 fold) (compare lanes 14–16 to lane 10, Fig. 7*B*), with detection of a full-length product at the highest concentration of Ac-RPA indicating complete replacement of the 60 nt downstream primer in a subset of substrates. Traces provided next to the gel images allow for better visualization in alterations to synthesis patterns in the presence of the highest concentration of either Um-RPA or Ac-RPA. Our data reveals that in the presence of Ac-RPA synthesis and strand displacement synthesis activities of Pol δ are enhanced, potentially leading to the formation of longer flaps during OFM.

### Ac-RPA inhibits FEN1 binding and cleavage on shorter flaps

Most of the flaps generated through Pol δ strand displacement synthesis during Okazaki fragment maturation (OFM) are cleaved by FEN1 using the short flap pathway. However, a small number of flaps are processed through the long flap pathway that involves both FEN1 and Dna2. It is unclear how the cell regulates processing through the short vs long flap pathway. However, there are a few instances where FEN1 cleavage is inhibited in the cell: through the presence of a blocked 5′ flap (either due to secondary structure or due to binding of another protein to the flap base) and modulation of the protein by lysine acetylation or phosphorylation (57, 58, 59). Additionally, the binding of RPA to the 5′flap inhibits FEN1 cleavage. Given that lysine acetylation has opposing impact on substrate binding by FEN1 (inhibitory) (58) and Dna2 (stimulatory) (43), we explored the impact of unmodified and acetylated proteins on flap binding and processing.

**Figure 8:**
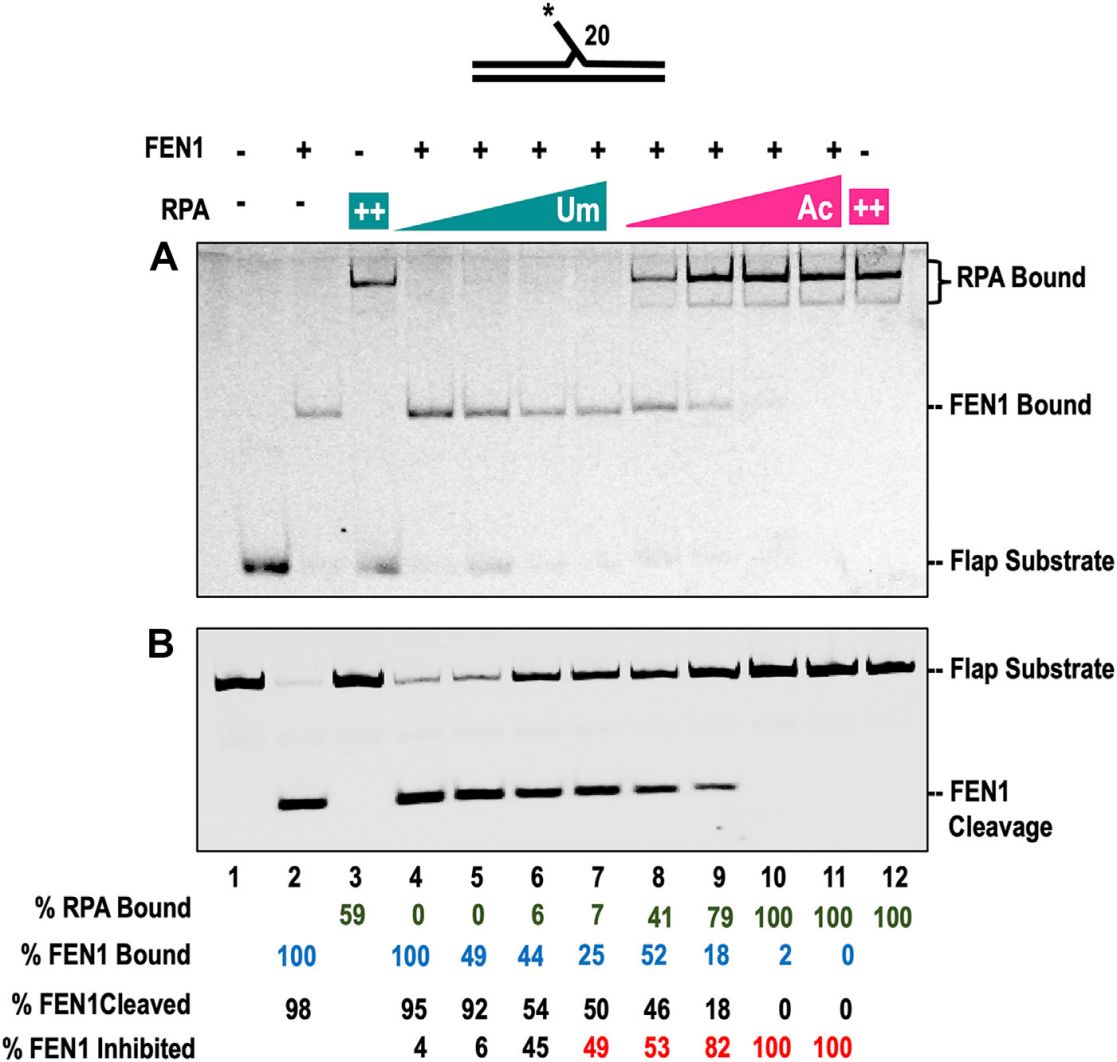
**Ac-RPA inhibits FEN1 binding and cleavage on short flaps.***A*, the dynamic binding of RPA (Um and Ac) to a 5 nM short double 5′ flap (20 nt) was assessed using an electrophoretic mobility gel shift assay (EMSA). 50 nM of FEN1 was incubated with increasing concentrations (5, 10, 25, and 50 nM) of Um-RPA (lanes 3–6) or Ac-RPA (lanes 8–11) as described in the Materials and Methods. Substrate-bound proteins were visualized and indicated on a native PAGE gel. *B*, cleavage activity of FEN1 (0.5 nM) on a 5 nM short double 5′ flap (20 nt) substrate was measured in the presence of increasing concentrations of (5, 10, 25, and 50 nM) of Um-RPA and Ac-RPA. The asterisk indicates the position of the IR label, and FEN1 cleavage products are indicated on the gel.

On a short 20-nt 5′ flap substrate, we anticipated dynamic RPA binding characterized by rapid association and dissociation kinetics. To evaluate this, we pre-bound FEN1 to the 20-nt flap substrate and titrated in either unmodified or acetylated RPA, assessing both RPA and FEN1 binding, as well as FEN1 cleavage activity. FEN1 exhibited high-affinity binding to the flap substrate (lane 2, Fig. 8*A*), whereas Um-RPA displayed only ∼53% binding at the highest concentration tested (lane 3, Fig. 8*A*). In contrast, Ac-RPA demonstrated complete binding to the substrate (lane 12, Fig. 8*A*), consistent with our previous binding data (Fig. 4).

When Um-RPA was titrated in the presence of pre-bound FEN1, we observed no substantial increase in RPA binding, but a reduction in FEN1 binding was evident (lanes 4–7, Fig. 8*A*). This partial displacement of FEN1 and transient binding by Um-RPA resulted in an approximately 49% decrease in FEN1 cleavage activity (compare lanes 4–7 to lane 2, Fig. 8*B*). Conversely, titration of Ac-RPA led to a marked increase in RPA binding and a near-complete loss of the FEN1-bound complex (lanes 8–11, Fig. 8*A*), which corresponded with a significant enhancement in FEN1 cleavage activity (lanes 8–11, Fig. 8*B*). These results suggest that RPA acetylation facilitates its binding to short flap substrates, leading to displacement of FEN1 and modulation of its enzymatic activity. This finding highlights a critical regulatory role for RPA acetylation in flap processing by impeding the short flap pathway.

### Ac-RPA alters the cleavage pattern of Dna2 and stimulates helicase activity on short flaps

**Figure 9:**
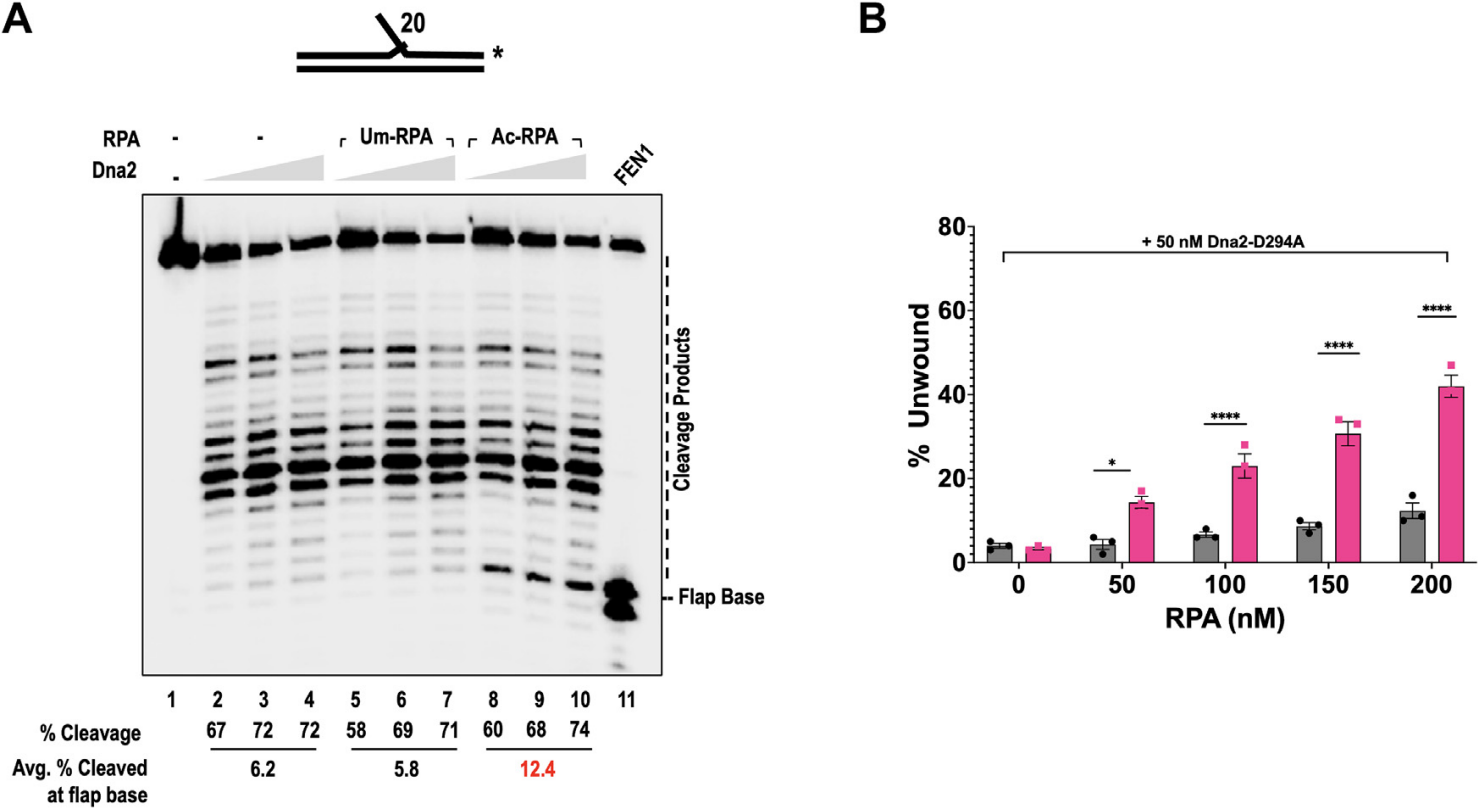
**Ac-RPA alters the cleavage pattern and stimulates the helicase activity of Dna2.***A*, increasing concentrations of Dna2 (50, 100 and 200 nM) were incubated with 2.5 nM 3′ labeled short 5′flap (20 nt) substrate, either in the absence of RPA (lanes 2–4), presence of 50 nM Um-RPA (lanes 5–7), or presence of 50 nM Ac-RPA (lanes 8–10). Sequential cleavage pattern was visualized by electrophoresing the reaction products on a 12% denaturing PAGE gel. To determine the location of the flap base, 100 nM FEN1 was incubated with the substrate (lane 11). Cleavage products and flap base are indicated on the gel and the substrate indicated above the gel. The asterisk indicates the location of the 3′-^32^P label. *B*, increasing concentrations of Um-RPA or Ac-RPA (50, 100, 150, 200 nM) were incubated with 5 nM 5′ IR-labeled short 5′flap (20 nt) substrate. The data for the helicase reactions are graphically represented as Mean + SEM (the average of three independent experiments). Significant two-way ANOVA effects denoted ∗ *p* < 0.01, ∗∗∗∗*p* < 0.0001.

Previous studies outlining the long flap pathway have shown that the endonuclease/helicase Dna2 can displace RPA while cleaving at multiple sites on the primer flap (18, 60, 61). Most of the work done on this pathway has explored the interaction between Dna2 and RPA, mostly on longer flapped substrates (∼ over 30 nt), because RPA can stably bind to flaps longer than 28 nt (20). However, Dna2 can also function efficiently on shorter flap substrates, ranging from 15 to 25 nt in length, independent of RPA (20). In this experiment, we reasoned that if acetylation of RPA promoted more stable binding to shorter flaps, then this interaction would still necessitate cleavage by Dna2. Therefore, we evaluated the influence of RPA acetylation on Dna2 activity when acting on short flaps. In the presence and absence of a fixed concentration of Um-RPA and Ac-RPA prebound to a 20 nt double flap, varying concentrations of Dna2 were titrated. Comparing the cleavage profile of Dna2 in the absence (lanes 2–4, Fig. 9) and presence of Um-RPA (lanes 5–7, Fig. 9) revealed that the efficiency of cleavage is unperturbed when RPA is pre-bound to the 20 nt flap substrate. However, we did not observe a change in the pattern of cleavage as has been previously reported to be the case for yeast Dna2 on longer flaps (62). When we compared the cleavage profile of Dna2 in the presence of Ac-RPA (lanes 8–9, Fig. 9*A*), we observed a 2-fold accumulation of cleavage products around the base of the flap showing that lysine acetylation of RPA directs Dna2 to cleave at the flap base, accounting for 12% of all cleaved products, when acting on a short flap. Cleavage at the base of the flap was identified by having a FEN1 alone control lane (lane 11, Fig. 9*A*).

It has been demonstrated that yeast RPA, particularly the 70 kDa domain, enhances the helicase activity of yeast Dna2 (63). To investigate the effects of unmodified *versus* acetylated RPA on Dna2's helicase activity, we used the nuclease-deficient D294A mutant in our experiment (Fig. 9B). We assessed helicase activity using a 20 nt flap substrate to examine the relationship between helicase and nuclease functions. Our results revealed that while Um-RPA did not substantially increase Dna2 helicase activity at the concentrations used, Ac-RPA led to ∼ 3.4-fold increase in helicase activity in the Dna2 mutant (Fig. 9*B*). The increase in the observed unwound product can be attributed to a combination of increase in Dna2 helicase activity as well as increased melting function by Ac-RPA. This enhanced helicase activity corresponds with greater cleavage observed at the flap base, suggesting that acetylated RPA enables Dna2 to bypass the need for FEN1 activity in creating a ligatable nick at the flap base.

## Discussion

In the present study, we demonstrate that RPA is acetylated *in vitro* by acetyltransferase p300. This was confirmed by mass spectrometry where six acetylated lysine residues (K163, K167, K259, K489, K502, and K577) on the RPA1 subunit were identified (Fig. 1). Moreover, p300 was also able to modify RPA1 within a cellular context. While previous studies have shown both Gcn5 and PCAF as capable of acetylating RPA1, our studies now show that in addition to the reported KATs, p300 is also a modifier of RPA. Assessment of cellular factors that impact the acetylation status of RPA revealed the cell cycle phases influenced this modification, with acetylation of RPA1 increasing through the S phase of the cell cycle and being maintained through the G2/M phase (Fig. 2). It appears that acetylation might be reset during the G1 phase, suggesting a role for this dynamic modification to regulate the protein within the cell.

Acetylation of proteins involved in DNA metabolism such as FEN1 and Dna2 have been previously reported to increase on UV-induced DNA damage (43). Similarly, we observed an increase in acetylation of RPA1 upon DNA damage caused by UV damage, despite no change in total levels of RPA (Fig. 3). This suggests that acetylation of RPA1 does not affect RPA stability. The increased acetylation of RPA on UV damage could be attributed to various factors—increased acetyltransferase (KAT) activity or decreased deacetylase (KDAC) activity, either globally or specifically for RPA. Lysine crotonylation and ubiquitination are other PTMs associated with RPA on exposure to UV damage. This suggests that RPA might be subject to competing modifications within the cell, which could differentially regulate its function. Importantly, we also demonstrated that RPA directly associated with replication and/or damaged forks is acetylated. Thus, this modification may play a role in how RPA interacts with its substrate. Additionally, given RPA’s interaction with numerous protein partners during replication and repair, acetylation may impact its interactions within these biological pathways. Post-translational modifications such as this could be a means of fine-tuning the activity of a multifaceted protein such as RPA that has roles in multiple pathways. However, more studies are needed to tease apart these acetylation-based alterations in protein interactions.

For most proteins, lysine residues near the DNA-binding domains generally enhance binding activity while those within the DNA binding domains usually repress binding efficiency to the specific DNA substrate (64, 65). Four of the six lysine sites identified in our study lie within the DBDs of RPA1. K259 resides within DBD-A, while K489, K502, and K577 are in DBD-C. The remaining two sites (K163 and K167) lie in the linker region between DBD-F and DBD-A. Acetylation of lysine residues regulating DNA binding activity as a function of proximity to the DNA binding domains is not unique to RPA (64, 65). Typically, addition of an acetyl group to a positively charged lysine residue results in the neutralizing the charge on the amino acid, and in turn reducing affinity of the protein to the negatively charged DNA substrates. However, *in vitro* characterization of acetylated RPA1 revealed that acetylation increases its ssDNA binding affinity (Fig. 4) and its dsDNA melting property while reducing its ssDNA annealing function. This is likely due to alterations in the conformation or dynamics of the RPA1 subunit upon acetylation, which could potentially also impact interaction with both ssDNA as well as its other protein interacting partners. Acetylation of p53 has been reported to open its normally closed conformation and increase DNA binding, thereby affecting its transcriptional activity (66). Similar to p53, we propose that acetylation of RPA may result in a more “open” conformation of the DNA-binding domains on RPA1, providing more access to DNA. This could explain the increased affinity for binding ssDNA, especially to shorter lengths of oligos, as well as the “tighter”/stronger binding to ssDNA, as shown by the competitor assay (Fig. 5). This has been observed for various other cellular proteins including p53 (66), E2F1 (67), STAT3 (68), GATA1 transcription factor (69), AP endonuclease (70), p50 and p65 (NF-κB) (71) among many others, where acetylation improves their DNA binding properties.

Acetyltransferase p300 has been shown to acetylate both histones and non-histone replication/repair associated proteins such as Pol δ (72), Pol β (73), FEN1 (58), Dna2 (43), p53 (74), PCNA (75), and WRN (76, 77) among others, thereby regulating their function. Acetylation of PCNA prevents its excessive accumulation on chromatin (75), while p53 acetylation influences its activation and stability in the cell (74) and WRN acetylation impacts both its stability and nuclear trafficking (76, 77). The number of p300 acetylated sites on RPA1 subunit is proportional to the increase in ssDNA binding affinity as shown from our RPA1 mutant data (Fig. S5). This suggests that acetylation of RPA1 at multiple lysine sites possibly causes a greater conformational change in the DBDs of RPA1 than at individual lysine acetylation sites. This further aids in our model of an “open” conformation of acetylated RPA1 that results in better access to DNA.

RPA association with ssDNA is crucial for all its biological functions. During DNA replication, unwinding of the DNA duplex results in the generation of ssDNA, which is efficiently coated by RPA, acting as an accessory factor for Pol δ (15). The strand melting properties of RPA aid in Pol δ synthesis, especially in regions that are hard to replicate such as those containing secondary structures, including triplex and G-quadraplex DNA (78, 79). During strand displacement synthesis, Pol δ gets rid of some proportion of the Pol α synthesized initiator primer. Due to lacking an exonuclease proofreading function, Pol α is considered error-prone compared to Pol δ and Pol ε. Increased strand displacement synthesis would allow for removal of a higher fraction or compete removal of the pol α synthesized primer. This region would be resynthesized by the proof-reading proficient pol δ, allowing for high fidelity duplication. Our biochemical assays revealed that in the presence of Ac-RPA, the synthesis as well as the strand displacement properties of Pol δ are enhanced (Fig. 7).

**Figure 10:**
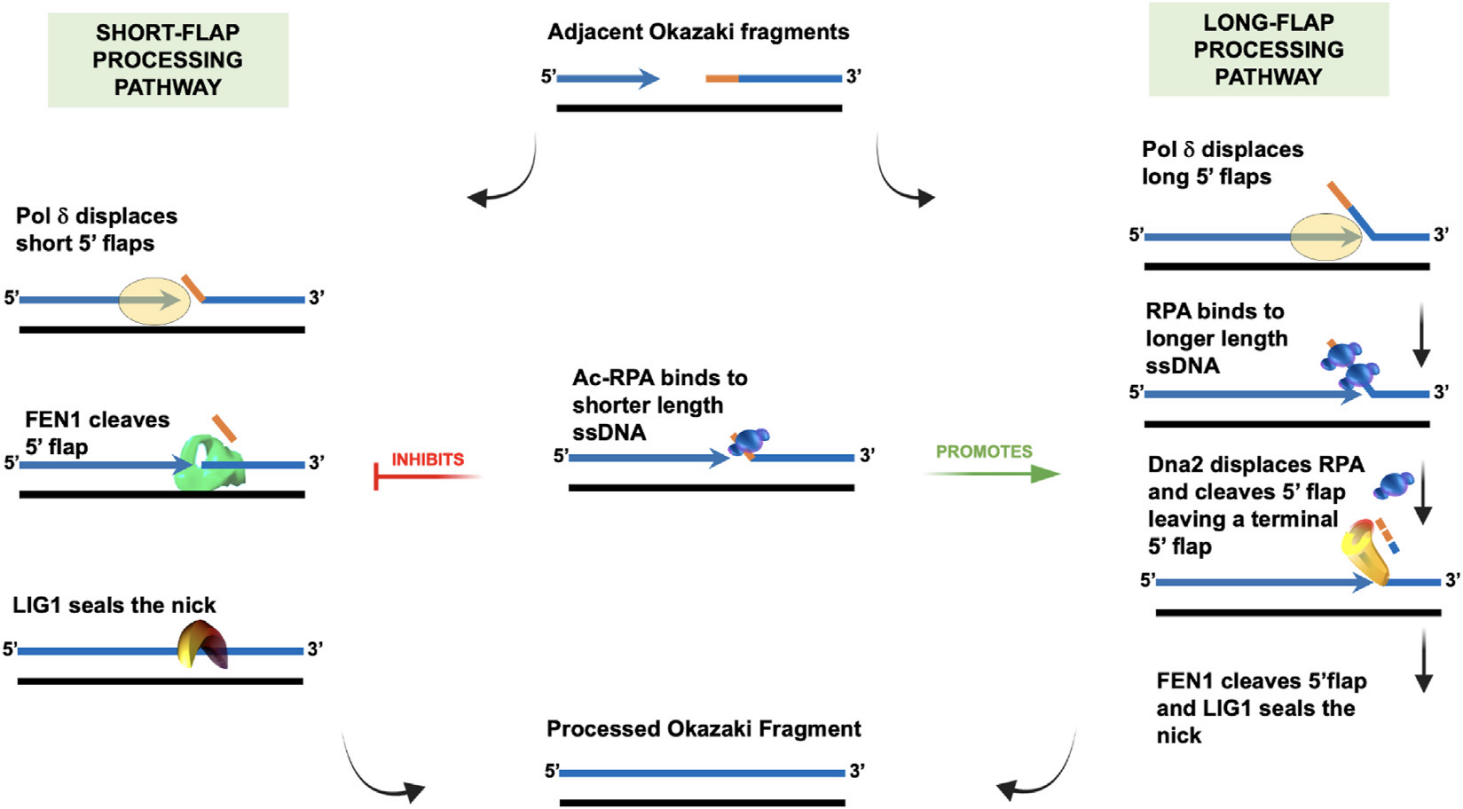
**Model for Ac-RPA governing the pathway choice for Okazaki fragment maturation.** The template is denoted in *black line*, Okazaki fragments in blue lines, and Pol α-synthesized initiator RNA/DNA primer in *orange line*. Different steps and functions of proteins involved in the Okazaki fragment maturation pathway are indicated. Binding of Ac-RPA to the Pol δ displaced short 5′ flaps prevents activity by FEN1 and thus inhibition of the short-flap processing pathway. Ac-RPA promotes processing of the bound flaps through the long flap pathway wherein the displaced flaps are processed first by Dna2, and the subsequently shortened flap is cleaved by FEN1 and ligated by LIG1.

Flaps displaced during OFM are efficiently cleaved by FEN1. However, acetylation of FEN1 inhibits its nuclease activity. We further show that Ac-RPA can stably coat shorter length flaps inhibiting FEN1 binding and cleavage (Fig. 8). Thus, the binding of Ac-RPA to the flap results in preferential processing of the flap through the long flap pathway. We found that in the presence of Ac-RPA the cleavage pattern of Dna2 is altered, displacing increased cleavage towards the base of the flap (Fig. 9). If Dna2 can cleave close to the flap base, then it can circumvent the need for FEN1 activity to create a ligatable nick. This alteration in Dna2 cleavage pattern can be explained by the increased helicase activity of Dna2 in the presence of Ac-RPA, allowing for cleavage toward the flap base (Fig. 9). The unwinding of the downstream primer may result from the combined effects of enhanced helicase activity of Dna2 in the presence of Ac-RPA as well as the increased melting properties of Ac-RPA. We did not detect any impact of Ac-RPA on the ligation efficiency of LigI (*data not shown*). Additionally, the functional properties of each of the OFM proteins (Pol δ, FEN1 and Dna2) are altered by p300-mediated acetylation. However, singular modification of RPA by acetylation can govern the switch from the short flap to the long flap pathway for OFM. Taken together, our data suggests that acetylation maybe a mechanism of promoting genomic stability by processing Okazaki fragments through the long flap pathway, which would result in longer stretches of initiator RNA-DNA primer being removed (proposed model, Fig. 10).

Our results showing increased ssDNA binding on RPA acetylation conflict with two studies that show the opposite effects. However, there are important experimental differences that explain the discrepancy. In the first study, yeast RPA was shown to be acetylated by NuA4 (Rfa1 K494) (80). Importantly acetylation occurred while RPA was bound to DNA. In these reactions, acetyl CoA was added to RPA that was prebound to a polydT83 ssDNA immobilized on magnetic beads along with NuA4. Upon the addition of Ac-CoA, they observed loss of RPA binding after 30- or 60-min suggesting that upon acetylation, RPA loses its binding ability to ssDNA (80). This observation was consistent with analysis of binding efficiency of 3KQ Rfa1 mutant (K259, K663, K494). In another study, human RPA1 lysine acetylation mutants (4KQ, acetylation mimic or 4KR, acetylation inhibitory – K259, K427, K463, K494) showed decreased ssDNA binding compared to the wild-type RPA, with the 4KQ mutant binding being impacted more significantly than the 4KR (81). With the exception of K259 all other sites identified in our studies are different from those identified in the other two studies. While *in vitro* acetylation reactions may be promiscuous, lysine acetylation mutants (be it KQ or KR) may impact protein structure and thus protein activity differentially from the native modification. While recombinant proteins can be generated wherein site-specific acetyl groups can be added using the nonsense-suppression methodology, this can be challenging when studying multiple lysine sites, since the reported yield of these recombinant proteins is not high enough to do extensive biochemical assays (82, 83). However, based on both prediction models (84) and sites identified in proteomic models (39) (Fig. S6), it is feasible that various lysine acetyltransferases could differentially acetylate multiple sites on RPA. This differential acetylation may subsequently alter RPA’s binding activity.

In conclusion, our studies involving p300-modified RPA have revealed that acetylation plays a crucial role in modulating RPA’s functions. Specifically, the acetylation of RPA was increased during the S phase of the cell cycle and maintained through the G2/M phases, and in response to UV-induced damage. Biochemical assays revealed acetylation enhances RPA’s affinity for ssDNA binding, reduces the off rate for dissociation, promotes the melting of dsDNA while concurrently diminishing its ability to anneal ssDNA. *In vitro* assays suggest that acetylation of RPA may function to play a regulatory role in how the cell processes the fragments in the lagging strand which have clear implications for replication fidelity. Furthermore, we propose that distinct lysine sites on RPA may undergo dynamic acetylation by different acetyltransferases, allowing RPA to finely regulate its multifaceted activities to achieve specific biological outcomes.

## Experimental procedures

### Recombinant proteins

Full-length human (h)RPA was expressed in the *E. coli* expression strain BL21(DE3) and purified as previously described (85). The constructs for all hRPA1 DNA-binding domains (DBDs) were generated using PCR in order to amplify specific regions of the RPA1 subunit. The PCR products were cloned into the pET28a vector, which introduced a six-histidine tag in frame at the C-terminus of each coding sequence. These constructs were expressed in *E. coli* BL21(DE3) cells and purified using Ni−NTA Superflow resin, as previously described (86). The catalytic subunit of p300 was expressed in *E. coli* expression strain BL21(DE3) and purified as previously described (87). Commercially available recombinant full-length p300 (#31124), catalytic domain of p300 (#31205), were purchased from Active Motif, Carlsbad, CA. FEN1 (88), Dna2 (89), Dna2-D294A (90) and Pol δ (72) were expressed and purified as previously described.

### Mass spectroscopy analysis

Tandem mass spectra for *in vitro* acetylated full-length RPA and RPA1 mutants were acquired in a data-dependent fashion using an LTQ-Orbitrap Velos mass spectrometer with XCalibur 2.2 SP1 software. The analysis employed a top-15 MS/MS method, with a dynamic repeat count set to one and a repeat duration of 30 s. The enzyme specificity was set to endoproteinase Lys-C, allowing up to two missed cleavages. High-quality peptide identifications were defined by cross-correlation (Xcorr) values of ≥1.5, delta CN values of ≥0.10, and precursor accuracy measurements within ±3 ppm for at least one injection. The mass accuracy for precursor ions was set to ±10 ppm, while for product ions it was ±0.8 Da. Carboxamidomethyl cysteine was designated as a fixed modification, with oxidized methionine and acetylated lysine residues allowed as dynamic modifications. Acetylated peptides were classified based on gene ontology (GO) annotations provided by Uniprot.

### Oligonucleotides

Synthetic oligonucleotides, including those containing 5′ biotin conjugation or 5-TAMARA label, were purchased from Integrated DNA Technologies (IDT). Biotinylated oligomers were used in BLItz assays to allow for binding to the streptavidin biosensors (Forte Biosciences, CA). Oligomers used in biochemical assays were either labeled with radiolabeled (^32^P) or fluorescently labeled (5′-TAMARA). Radiolabeling was performed on the 5′ end of oligonucleotides using [γ-^32^P] ATP [6000 μCi/mmol] (PerkinElmer) and polynucleotide kinase (Roche Applied Science) as previously described (91). Oligomer lengths and sequences (in the 5′-3′ orientation) are provided in Table S1.

### *In vitro* acetylation

Recombinant human RPA was acetylated by incubating it in 1X histone acetyltransferase (HAT) buffer [50 mM Tris-HCl (pH 8.0), 10% (v/v) glycerol, 150 mM NaCl, 1 mM dithiothreitol, 1 mM phenylmethylsulfonyl fluoride, 10 mM sodium butyrate] with either the full length (or catalytic domain) of p300, full length GCN5, full length PCAF and acetyl CoA in a 1:1:10 ratio [RPA (full length or RPA1mutants): acetyltransferase: acetyl CoA] for 30 min at 37°C. The unmodified RPA (Um-RPA) control and the control with Um-RPA and p300 (Um-RPA + AT) were treated similar to the acetylated RPA (Ac-RPA). For autoradiography, *in vitro* acetylation reactions were performed using 0.1 μCi [^14^C] acetyl coenzyme A (PerkinElmer Life Sciences). The unmodified and acetylated forms of RPA were separated on a 4 to 15% SDS PAGE gel. After electrophoresis, the gels were stained with Coomassie brilliant blue (CBB), imaged and subsequently dried for autoradiography analysis.

### Mammalian cell culture

Human embryonic kidney (HEK293T) cells (CRL-1573) were purchased from ATCC and cultured in Minimum Essential Media (MEM) supplemented with 10% fetal bovine serum (FBS), 2 mM L-glutamine, and 1% penicillin/streptomycin. HCT116 parent wild-type cells and HCT116 p300 knockout D10 clone were purchased from Cancer Research UK Cambridge Institute and cultured in McCoy’s 5A medium supplemented with 10% FBS, 2 mM L-glutamine and 1% penicillin/streptomycin. Cells were incubated at 37 °C in a humidified 5% CO_2_ environment and grown to approximately 80% confluency before the next passage or further experiments. The EP300 cDNA plasmid in pcDNA3.1-p300 was a gift from Warner Greene (Addgene plasmid # 23252) (92). For transfection experiments, 0.7 × 10^6^ cells (in 3 ml) of either HEK293 or HCT116 p300^-^ in the respective media were seeded in a T-25 flask and 24 h later was transfected with EP300 plasmid construct (1 μg or 2.5 μg) using Lipofectamine 3000 according to the manufacturer’s protocol (Invitrogen). Following 24 h of transfection, the cells were washed with 1X PBS, harvested, and lysed in RIPA buffer containing 10 mM sodium butyrate.

#### Cell synchronization

HEK293T cells were arrested in S phase by treating them with 2.5 mM thymidine for 17 h before harvest. Cell lysates were prepared as described above.

### Immunofluorescence

HEK293Tcells were plated in an 8-well chamber slide at 1 × 10^4^ cells/well and allowed to adhere overnight. Cells were treated with 10 μM SAHA or vehicle control (0.02% DMSO) for 2 h. After treatment, cells were washed twice with PBS, fixed in 4% paraformaldehyde for 30 min, and permeabilized with 0.25% Triton X-100 in PBS for 15 min at room temperature. The slide was blocked with PBST containing 1% BSA and 10% normal goat serum for 1 h at room temperature then incubated overnight at 4oC with RPA_Ac-K163 antibody_ (1:200 in blocking buffer). After primary antibody incubation, the slide was washed 3 × 5 min in PBST and incubated for 30 min with 1:1000 dilution of Goat anti-Rabbit IgG (H+L) Cross-Adsorbed Secondary Antibody, PE-Alexa Fluor 488 (ThermoFisher Scientific) and washed again with PBST. DNA was stained with 1ug/ml DAPI in PBS for 15 min, washed to remove excess DAPI and the slide was mounted with Prolong Diamond Anti-Fade Mountant (ThermoFisher Scientific). Images were captured with an EVOS FL Auto2 microscope.

### Cell cycle and AcRPA detection

HEK293T cells were plated in six-well dishes at 3 × 10^5^ cells/well and allowed to adhere overnight. Cells were treated with 10 μM SAHA or vehicle control (0.02% DMSO) for 2 h. After treatment cells were washed twice with PBS, trypsinized and washed off the plate with PBS containing 1% FBS and 1 mM EDTA. After centrifugation at 400*g* for 3 min, cells were washed once with 1% BSA in PBS then fixed in 4% paraformaldehyde at room temperature for 15 min and permeabilized with 0.25% Triton X-100 or a saponin-based buffer (BD Perm/Wash Buffer, BD Biosciences). Cells were incubated overnight at 4 °C with RPA_Ac-K163_ antibody (1:200 in BD Perm/Wash Buffer), washed with BD Perm/Wash Buffer, then incubated for 30 min with 1:1000 dilution of Goat anti-Rabbit IgG (H+L) Cross-Adsorbed Secondary Antibody, PE-Alexa Fluor 488 (ThermoFisher Scientific) and washed with BD Perm/Wash Buffer. After antibody incubations, DNA was stained with Guava Cell Cycle Reagent (Cytek) for 30 min, and data was acquired on a Guava easyCyte flow cytometer.

### EdU incorporation and AcRPA detection

HEK293T cells were plated in six well dishes at 3 × 10^5^ cells/well and allowed to adhere overnight then treated with 10 μM SAHA or vehicle control (0.02% DMSO) for 2 h and labeled with EdU for 30 min. After treatment cells were washed twice with PBS, released from the plate with trypsin and collected with PBS containing 1% FBS and 1 mM EDTA. Cells were sedimented at 400*g* for 3 min and washed once with 1% BSA in PBS. EdU was detected with the Click-iT EdU Flow Cytometry Assay Kit (Molecular Probes) according to the manufacturer’s protocol. Briefly, cells were fixed in Click-iT fixative for 15 min at room temperature and permeabilized with Click-iT saponin-based permeabilization and wash reagent. Click-It reaction was carried out at room temperature for 30 min, after which cells were washed in Click-iT saponin-based permeabilization and wash reagent. Cells were then incubated overnight at 4 °C with RPA_Ac-K163_ antibody (1:200 in Perm/Wash Buffer), washed with Perm/Wash Buffer, then incubated for 30 min with 1:1000 dilution of Goat anti-Rabbit IgG (H+L) Cross-Adsorbed Secondary Antibody, PE-Alexa Fluor 647 (ThermoFisher Scientific) and washed with Perm/Wash Buffer. After antibody incubations, data were acquired on a Guava easyCyte flow cytometer.

### DNA damaging agent treatment

For hydroxyurea (HU) treatment, cells were treated with 4 μM HU for 1, 3, and 6 h. For methyl methanesulfonate (MMS) treatment, cells were treated with 2 mM MMS for 4, 8, and 12 h. For ultraviolet (UV) treatment, cells were washed and maintained in warm 1X PBS during UV exposure. UV radiation of 10 J/m^2^ was administered at 254 nm (UV-C) using a CL-1000 UV crosslinker (UVP, CA). Media was then replaced in the dishes, and cells were incubated for 4, 8, and 12 h before harvesting the cell lysate.

After specified hours of treatment, cells were washed thrice with 1X PBS and lysed in RIPA buffer containing 10 mM sodium butyrate, lysates were quantified and further used in immunoprecipitation and western blot experiments. Dimethyl sulfoxide (DMSO) was used as the untreated control for HU, MMS and ETP experiments. For the UV experiment, untreated cells were handled in a similar manner to the treated cells with the exception of exposing cells to UV.

### Generation of RPA1_Ac-K163_ antibody

The anti-RPA1_Ac-K163_ antibody was generated and purified Genmed Synthesis Inc. Briefly, two peptides were synthesized, one specifically for antibody production and affinity purification (C+AYGASK(ac)TFGKAAGP) and a control peptide for affinity purification (C+AYGASKTFGKAAGP). These peptides were purified to get >75% purity and were conjugated to keyhole limpet hemocyanin (KLH) carrier protein. This was then injected into two rabbits to generate the antibody. The antibody was subsequently purified using affinity columns and ELISA was performed to confirm specificity of the antibody (Fig. S2*A*). Two independent batches of the antibody were generated and utilized in our studies, both of which exhibited similar specificity to acetylated RPA as described.

### Isolation of proteins on nascent DNA (iPOND) assay

HEK293T cells were subject to iPOND assay using a previously published protocol (93). Cells were synchronized in the S phase or treated with different damaging agents as outlined above. Synchronized or treated HEK293T cells (1 × 10^8^) were labeled with 20 μM EdU for 15 min alone or chased in the presence of 25 μM thymidine. Labeled cells were fixed and subjected to click-chemistry as described in the protocol. Replication proteins were eluted under reducing conditions by boiling in 2X SDS-sample buffer for 60 min. All buffers in the assay contained 10 mM sodium butyrate to prevent deacetylase activity and subsequent loss of acetylation signal from RPA1. Samples were then analyzed by western blotting as indicated in figure legend (Figs. 2*D* and 3*C*).

### Co-immunoprecipitation

Immunoprecipitation was performed using the protocol described in the Dynabeads protein G manual (Thermo Fisher Scientific) with minor modifications. Briefly, 20 μl of antibodies to acetyl-lysine or control IgG were prebound to 1 mg of HEK293 whole cell extract from different DNA damaging treatments and cell cycle phases with 200 μl of 1X PBST for 1 h at room temperature with end-over mixing. Dynabeads (50 μl) were prepared by magnetic separation to remove the buffer and cell lysates were added to the beads and incubated with end-over mixing for 30 min at room temperature. The Dynabeads–Ab–antigen complex was then washed thrice with 200 μls of washing buffer and separated on a magnet between washes. Elution was carried out using 20 μl Elution buffer and 20 μl of premixed 2X NuPAGE LDS sample buffer with NuPAGE sample reducing agent followed by heating the samples at 70 °C for 10 min. The immunoprecipitate was separated on the magnet, and the supernatant was separated on precast 7.5% TGX gels (Bio-Rad). Western blot analysis was performed with anti-RPA1 antibody (Millipore # MS-692-P).

### Western blot analysis

Synchronized or treated human embryonic kidney (HEK293T) cells were lysed in RIPA buffer (Thermo Fisher Scientific # 89901) containing 10 mM of sodium butyrate. Protein concentration was determined using BCA Protein Assay (Pierce). Cell lysates (30 μg) were separated on precast 4 to 15% or 7.5% SDS-polyacrylamide Criterion gels (Bio-Rad) and transferred to polyvinylidene difluoride (PVDF) membranes (Bio-Rad). The following primary antibodies were used at 1:1000 dilution in overnight incubations at 4 °C: RPA1_AC-K163_, RPA1, p-RPA2, RPA2, p-p53, p-Chk2, p-H2AX and GAPDH. Secondary antibody (HRP-conjugated anti-rabbit IgG, anti-goat IgG or anti-mouse IgG) was added and incubated at room temperature for 1 h at 1:3000 dilution. The anti-RPA1 (mouse monoclonal) was purchased from Thermo Fisher scientific (Waltham, MA). The anti-RPA2 (rabbit polyclonal) and anti-GAPDH were purchased from Santa Cruz Biotechnology . Anti-p-Chk2 (2197), anti-p-H2AX (9718), anti-p-p53 (9286) and all secondary antibodies were purchased from Cell signaling while anti-p-RPA2 (ab109394) was purchased from Abcam. Blots were visualized using Cytiva ECL western blotting detection reagent (Cytiva) and GE ImageQuant LAS4000 mini-Imager. Blots were quantified by densitometry using LI-COR Image Studio Lite Ver 5.2.

### BLItz analysis

The BLItz binding system was equipped with a Dip and Read Streptavidin (SA) biosensors (ForteBio). BLItz binding assays were performed to measure binding between unmodified RPA (Um-RPA), RPA in the presence of acetyltransferase p300 (Um-RPA + AT), or acetylated RPA (Ac-RPA) with biotinylated ssDNA substrates. 10 nM of the biotinylated oligo of various lengths (20, 24, 28, 32, and 45 nt) was immobilized by the Streptavidin (SA) biosensor for 120 s. The Streptavidin (SA) biosensor immobilized by biotinylated oligo was dipped into 4 μl of RPA (Um or +AT or Ac) solution at different concentrations (31.25, 62.5, 125, or 250 nM) for 150 s association, and 150 s dissociation in 1X HAT buffer. The real-time wavelength shift was recorded and analyzed by ForteBio software.

### Electrophoretic mobility gel shift assays

Binding efficiency of Um-RPA, RPA + AT and Ac-RPA to 20, 25, 29 and 32 nt ssDNA were assessed using electrophoretic mobility gel shift assays. Five nanomolar of substrate was incubated with increasing concentrations (1, 2.5, and 5 nM) of either unmodified hRPA (Um-RPA) or acetylated hRPA (Ac-RPA) and incubated for 10 min at 37 °C in EMSA buffer consisting of 50mMTris-HCl (pH 8.0), 2 mM dithiothreitol, 30 mM NaCl, 0.1 mg/ml bovine serum albumin, and 5% glycerol. The reactions were loaded on pre-run 6% polyacrylamide gels in 1X Tris-borate EDTA (TBE) buffer. Gels were subjected to electrophoresis for 1 h 45 min at constant 180 V. EMSA’s assessing binding efficiency of RPA1 mutants utilized 25 nM of 5′TAMARA labeled 30 nt ssDNA incubated with increasing concentrations of Um- or Ac-RPA1 mutants (25, 50, 100, 150 nM). Acetylation sites on the mutants were confirmed by mass spectrometry analysis.

### Competitor assay

Similar to the electrophoretic mobility gel shift assay, the binding efficiency of Um-RPA and Ac-RPA to radiolabeled substrates (24 nt and 28 nt) in the presence of varying concentrations of a cold competitor 28 nt oligomer were assessed. Five nanomolar of radiolabeled substrate was pre-incubated with 100 nM of hRPA (Um-RPA and Ac-RPA) at 37 °C for 2 min. To this reaction a competing non-radiolabeled oligomer (28 nt) was added at 100-, 250-, 500-, and 1000-fold excess of radiolabeled primers and the reactions and further incubated for an additional 8 min at 37 °C. Reactions were then loaded and electrophoresed similar to conditions described above.

### Strand annealing and melting assay

To assess the annealing efficiency of RPA, increasing concentrations of either Um-RPA or Ac-RPA (10, 25, 50, 75, 100, and 150 nM) were incubated with 25 nM of a 37 nt 5′TAMRA-labeled template and 50 nM of its unlabeled 57 nt complementary strand. Reactions were incubated at 37 °C for 30 min and loaded and electrophoresed similar to conditions described above. To assess the melting efficiency of RPA, increasing concentrations of either Um-RPA or Ac-RPA (10, 25, 50, 75, 100 and 150 nM) was incubated with a duplex substrate (TAMARA-labeled 37 nt annealed to 57 nt complementary template). Reactions were incubated at 37 °C for 30 min and loaded and electrophoresed similar to conditions described above. Reactions without RPA served as control and all values obtained for the assay was normalized to the no-RPA control.

### Pol δ synthesis assay

Polymerase activity by pol δ (100 nM) on a synthesis substrate (25U + 110T), and strand-displacement substrate (25U + 60D + 110T) was performed in the presence of varying concentrations of Um-RPA and Ac-RPA (50, 100, and 200 nM). The reactions were carried out in a reaction buffer containing 50 mM Tris-HCl (pH 8.0), 2 mM DTT, 2 μg/uL BSA, 2 mM ATP, 5 mM MgCl2, 1 mM dNTP mix, and 25 mM NaCl. Five nanomolar of each substrate was incubated with Pol δ and either UM-RPA or Ac-RPA at 37 °C for 10 min to a final volume of 20 μl. Reactions were terminated by adding 20 μl of 2X Termination Dye (90% formamide (v/v), 10 mM EDTA, 0.01% xylene cyanol and bromophenol blue) and boiled at 95 °C for 5 min. Samples were loaded onto a pre-run 12% polyacrylamide denaturation gel for 75 min at 80 W. The gels were exposed on a phosphor screen overnight and imaged using a Typhoon scanner. To quantify the relative difference in the synthesis by Pol delta in the presence of Um-RPA and Ac-RPA, synthesis products were quantified, using ImageQuant TL v8.1. The formula used to quantify overall synthesis was [(full-length product + intermediate products)/(full-length product + intermediate products + exonuclease products + synthesized substrate)∗100]. Full-length synthesis (110 nt product) was calculated as ([full-length)/(full length + intermediate products + exonuclease products + synthesized product)∗100]. Strand displacement product was calculated as (synthesis products past the pause point (50 nt)/(synthesis products + intermediate products + exonuclease products + synthesized substrate)∗100]. Additionally, traces comparing the Um-RPA and Ac-RPA at the highest concentrations are also displayed next to the gel images. Experiments were minimally triplicated, and representative gels are shown in the figures.

### FEN1 and RPA binding assay

To assess the impact of RPA binding to a prebound FEN1 flap substrate, 50 nM of FEN1 was bound to a 20 nt flap substrate for 5 min at room temperature in reaction buffer (50 mM Tris-HCl (pH 8.0), 2 mM DTT, 20 mM NaCl, 0.1 mg/ml BSA, 5% (v/v) glycerol and 20 μM EDTA), following which increasing concentrations of either unmodified or acetylated RPA (5, 10, 25 and 50 nM) were added to the tubes. The reaction was incubated at 37 °C for 10 min. RPA alone lanes contained 50 nM of either unmodified or acetylated protein.

The samples were loaded onto an 8% pre-run native polyacrylamide gel and electrophoresed at 250 V for 45 min. Gels were imaged using the previously outlined method. To determine the percentage of RPA or FEN1-bound flaps, the intensity of the RPA/FEN1-bound lanes was compared to the presence of unbound substrate and FEN1/RPA-bound substrates. The formula used to quantify this was [(RPA or FEN1 bound product)/(RPA bound product + FEN1 bound product + unbound substrate ∗100)]. Experiments were minimally triplicated, and representative gels are shown in the figures.

### FEN1 cleavage assay

The cleavage activity of FEN1 on a short flap (20 nt) was assessed in the presence of varying concentrations of human RPA. FEN1 (0.5 nM) was pre-bound to a 20 nt double flap substrate for 5 min at room temperature in reaction buffer (50 mM Tris-HCl (pH 8.0), 2 mM DTT, 20 mM NaCl, 0.1 mg/ml BSA, 5% (v/v) glycerol and 20 μM EDTA), following which increasing concentrations of either unmodified or acetylated RPA (5, 10, 25, and 50 nM) were added to the tubes. After the addition of RPA, 2 mM MgCl_2_ was added to the reaction and further incubated at 37 °C for 10 min. Reactions were terminated by the addition of 80 mM EDTA, 50% formamide (final volume), and 0.08% SDS as previously described and then boiled at 95 °C for 5 min. The samples were loaded onto an 8% pre-run native polyacrylamide gel and electrophoresed at 250 V for 45 min. Gels were imaged using the previously outlined method. The intensity of each band was measured, and the amount of cleaved product was calculated using the equation [(cleaved product)/(cleaved product + uncleaved substrate) ∗ 100]. Additionally, the percentage of cleavage inhibited by the presence of RPA was calculated by subtracting the number of cleaved products formed in the presence of RPA and FEN1 from the amount of product formed in the presence of FEN1 alone. Experiments were minimally triplicated, and representative gels are shown in the figures.

### Dna2 cleavage assay

To visualize the cleavage products resulting from the activity of the nuclease Dna2, a 3′ labelled 20 nt flap was pre-incubated with or without 50 nM Um-RPA and Ac-RPA for 5 min at 37 °C. Upon completion of incubation, varying concentrations of Dna2 (50, 100, and 200 nM) were added to a final reaction volume of 20 μl at 37 °C for 10 min. Reactions were performed in a buffer containing 50 mM Tris-HCl (pH 8.0), 2 mM DTT, 20 mM NaCl, 0.1 mg/ml BSA, 5% (v/v) glycerol, 2 mM ATP and 4 mM MgCl_2_ and terminated by the addition of 80 mM EDTA, 50% formamide (final volume), and 0.08% SDS. This was followed by boiling at 95 °C for 5 min. Samples were loaded onto a pre-run 12% polyacrylamide denaturing gel with 7M urea for 90 min at 80 W. The gels were exposed to a phosphor screen overnight and imaged using a Typhoon scanner. Using the Image Studio software, the intensity of each band was measured, and the amount of cleaved product was calculated using the equation [(cleaved product)/(cleaved product + uncleaved substrate) ∗ 100]. Furthermore, the percentage of cleaved products present at the flap base was calculated using the equation [(product at flap base)/(total cleaved product)∗100]. Experiments were minimally triplicated, and representative gels are shown in the figures.

### Dna2 helicase assay

Helicase activity of the nuclease dead mutant Dna2-D294A (50 nM) was assessed on a 5′ IR-labeled 20 nt flap substrate (5 nM), in the presence of increasing concentrations (50, 100, 150, and 200 nM) of Um-RPA and Ac-RPA. Reactions were performed in a buffer containing 50 mM Tris-HCl (pH 8.0), 2 mM DTT, 20 mM NaCl, 0.1 mg/ml BSA, 5% (v/v) glycerol, 8 mM ATP, and 4 mM MgCl_2_. The reactions were terminated using 6X helicase dye (50 mm EDTA, 0.9% SDS, 0.125% bromphenol blue, 0.125% xylene cyanole, 30% glycerol). After termination, samples were loaded on a pre-run 6% native PAGE and resolved by electrophoresis for 1.5 h at 150 V. Unwound products were calculated using the equation [(unwound product)/(unwound product + remnant substrate) ∗ 100].

## Gel analysis

Radioactive gels from all assays were dried, exposed to a phosphor screen, and analyzed using the Image Quant software as previously described (94). Assays containing the TAMRA-labeled oligonucleotides were visualized on a Typhoon FLA9600 Imager (GE Biosciences) using the preset laser excitation and emission settings, with a photomultiplier gain of 200 V. The percentage of RPA bound to substrate is defined as [bound/(bound + unbound)]. Fold change is defined as [bound (Ac-RPA/bound (Um-RPA)].

## Statistical analysis

Statistical significance was analyzed by was assessed by one-way ANOVA (for Fig. 2*B*), unpaired *t* test for Figure 2*C*, and two-way ANOVA with *post hoc* Šídák's multiple comparisons test (for Figs. 3, 6, and 7) using GraphPad Prism software (version 10.2.2).

## Data availability

All data are presented within the manuscript.

## Supporting information

This article contains supporting information.

## Conflict of interest

The authors declare the following financial interests/personal relationships which may be considered as potential competing interests: J.J. Turchi is a co-founder and CSO of NERx Biosciences and co-inventor on patents covering the compounds described in Figure S6.
