# Supplementary material for: Biochemical impact of p300-mediated acetylation of replication protein A: Implications for DNA metabolic pathway choice
Source: J Biol Chem. 2025 May 17;301(6):110250. doi: 10.1016/j.jbc.2025.110250 (PMC12182300; doi:10.1016/j.jbc.2025.110250)
Supplement: Supporting Information [file mmc1.pdf]

Supplementary Information

Biochemical Impact of p300-Mediated Acetylation of Replication Protein A: Implications for DNA Metabolic Pathway Choice

Onyekachi Ononye[1, §], Sneha Surendran[1,§], Tripthi Battapadi[1], Pamela VanderVere-Carozza [2], Olivia K. Howald[1], Athena Kantartzis-Petrides[3], Matthew R. Jordan[2], Diana Ainembabazi[2], Marc S. Wold[4], John J. Turchi[2], Lata Balakrishnan[1, ⌘]

[1]Department of Biology, School of Science, Indiana University Indianapolis, Indianapolis, IN, 46202, [2]Department of Pathology, Harvard Medical School, Boston, MA 02115, [3]Department of Medicine, Indiana University School of Medicine, Indianapolis, IN 46202, [4]Department of Biochemistry and Molecular Biology, Carver College of Medicine, University of Iowa, Iowa City, IA 52242.

*Running Title: Acetylation of Replication Protein A

[§]Co-first authors

[⌘]To whom correspondence should be addressed: Lata Balakrishnan, Department of Biology, Indiana University Indianapolis, Indianapolis, IN 46202, Phone: (317) 274 1290; Email: latabala@iu.edu

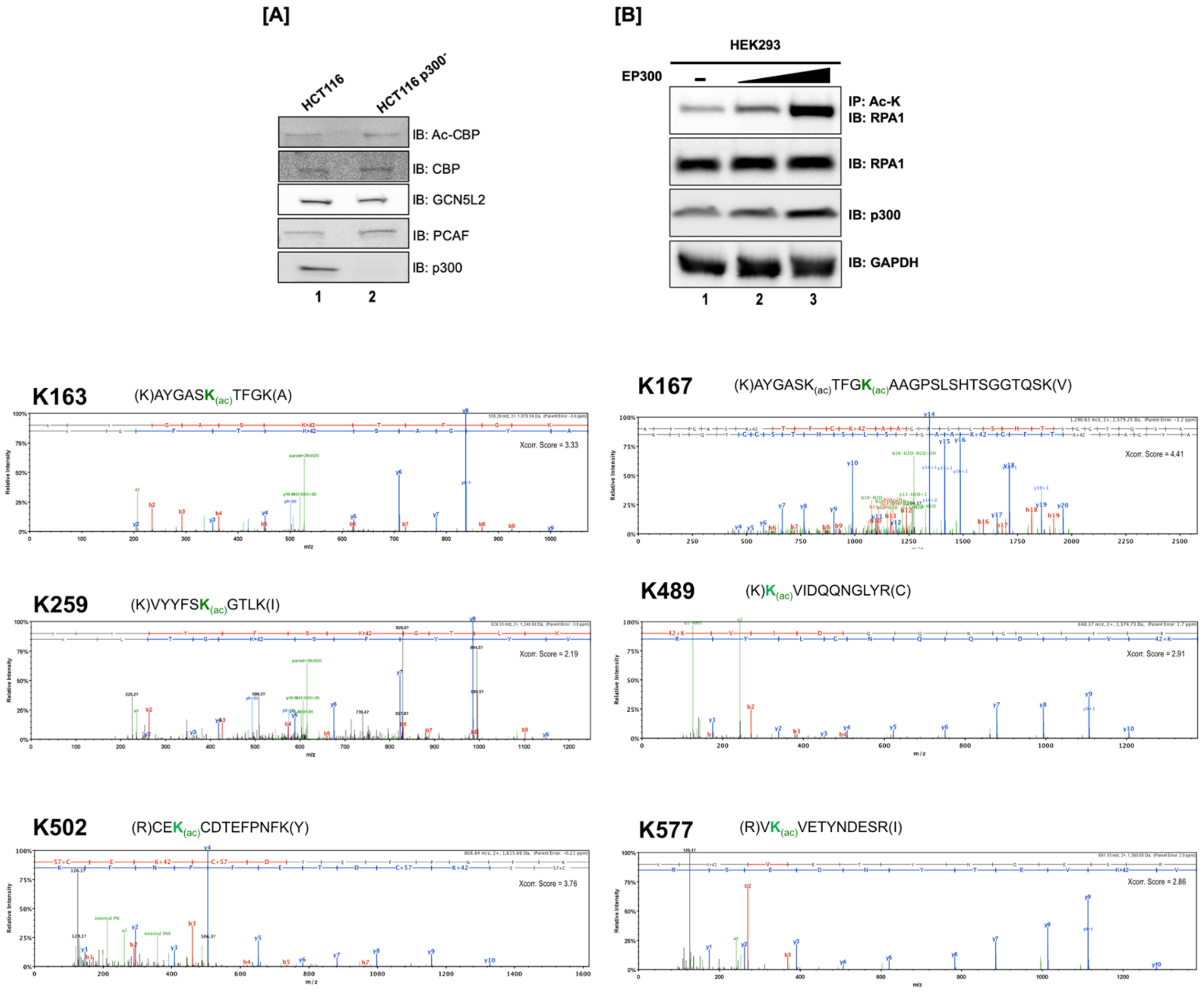

Supplementary Figure 1: (**A**) Immunoblot analysis of expression of specific KATs in wild-type HCT116 and HCT116 p300- cell lysates (**B**) Acetylation of RPA1 Subunit. IP-western blot analysis of RPA1 acetylation in HEK293 cells transfected with EP300 overexpression construct. **C**) MS/MS spectra for *in vitro* acetylated RPA1**.** Representative spectra for lysine acetylation sites on RPA1 annotated on Scaffold (Proteome Software, Portland OR). The b-ions are labeled in red and y-ions are labeled in blue. Neutral loss and other parent ion fragments are shown in green. Sequence of the acetylated peptide is denoted above the spectra with the acetylated lysine (K) highlighted in bold green font.

[A]

	Neat Serum			Affinity Purity Fraction (Acetyl)		
		Plates coated with acetyl peptide	Plates coated with control peptide		Plates coated with acetyl peptide	Plates coated with control peptide
A	1:100	3.042***	3.050***	1:100	3.039***	0.212
B	1:1000	3.034***	3.048***	1:1000	3.039***	0.039
C	1:10,000	3.026***	2.827***	1:10,000	1.153	0.033
D	1:100,000	1.093	0.538	1:100,000	0.183	0.022

[B]

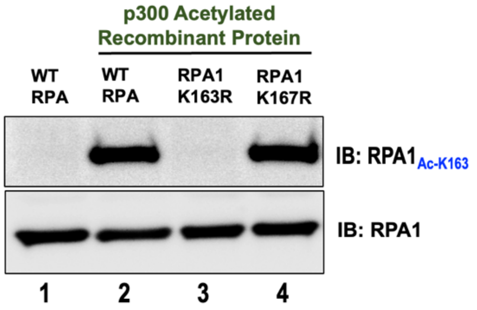

[C]

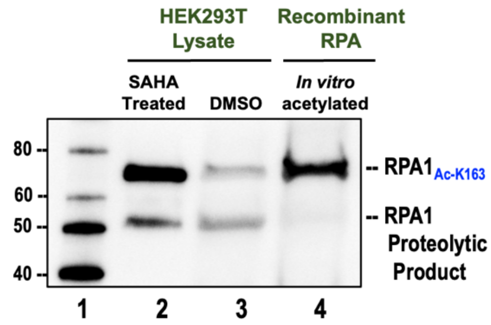

Supplementary Figure 2A: Testing the Specificity of RPA1$_{Ac\text{-}K163}$ antibody: ELISA Analysis of Custom Affinity Pure Sera: Antigens (free peptides) were coated on ELISA strips at 10 µg/ml in coating buffer. The unbound antigen was washed, and all remaining sites were blocked with buffer containing BSA. Anti-sera, including preimmune was diluted in 1:100, 1:1000, 1:10,000 and 1:100,000 and added in separate wells down the column. After 60 mins of antibody incubation, unbound antibodies were washed and the anti-rabbit IgG-HRP conjugate is added. The plates were washed again after 30 mins incubation. TMB substrate was then added, and color developed for 15 mins. The reaction (blue color) was stopped by the addition of acid (turns blue to yellow). The amount of yellow color (read at 450 nm with an ELISA reader) was directly proportional to the amount of antibody. Color is read in Absorbance or OD (Optical density) units of 0.000-2.000. Reading above 2.000 were not considered to be linear, since it displayed too much yellow color. This is represented by *** indicating excess color. Upon further antibody dilution, the absorbance was readable (Abs. < 2.000). This is represented as A450 nm. The ELISA assay showed that the antibodies were clearly detected in an antibody titre of 1:10,000 and 1:100,000 and blocking using the control peptide showed that the antibody was specific to the acetylated peptide. **(B)** Immunoblot analysis of p300-acetylated wild-type or acetylation mutant (KR) recombinant RPA proteins. The top blot was probed using the RPA1$_{Ac\text{-}K163}$ antibody and the bottom blot was probed using a RPA1 antibody **(C)** HEK293T cell lysates (from either untreated or treated for 24 hours with 10µM suberoylanilide hydroxamic acid (SAHA) to induce cellular hyperacetylation) were separated on a 4-20% gradient gel and subject to immunoblotting using the RPA1K$_{163ac}$ antibody (1:10,000 dilution). *In vitro* acetylated RPA (AcRPA) served as a positive control. The RPA1$_{Ac\text{-}K163}$ antibody recognized two products in the cell lysate, the 70 kDa RPA1 and the 55 kDa RPA1 proteolytic product.

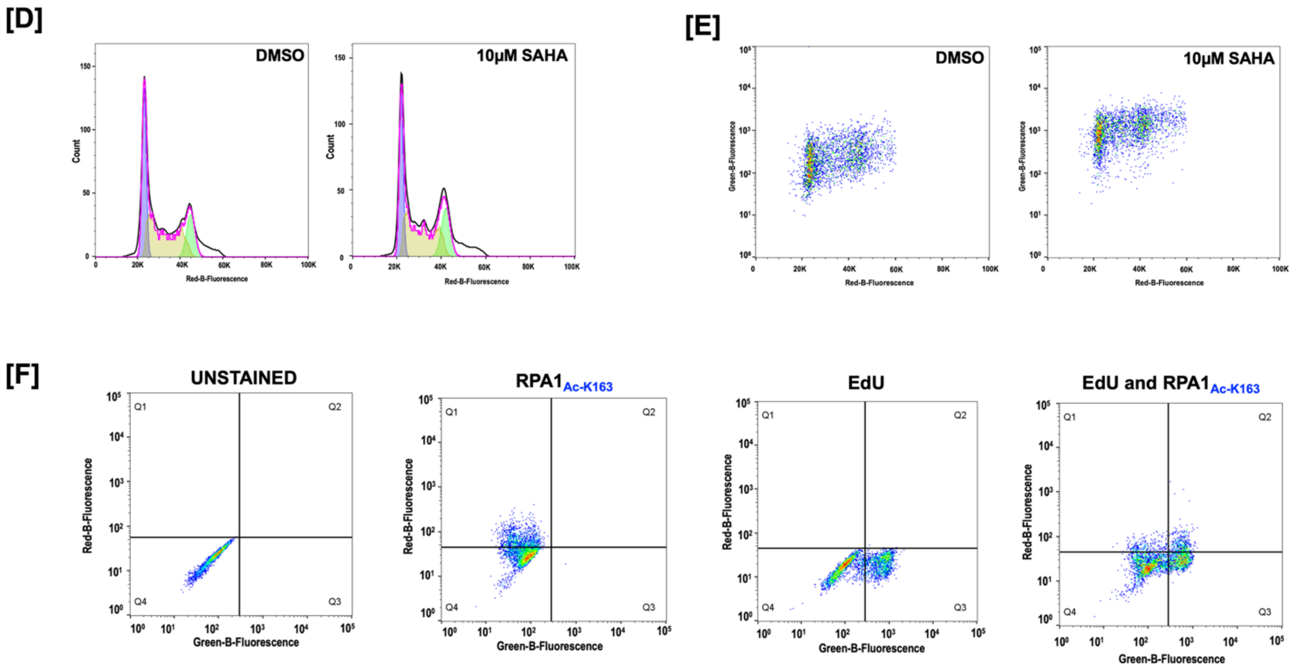

Supplementary Figure 2D-F: **Flow cytometry of HEK293T cells**. Data collected on a Guava EasyCyte and plotted with FlowJo software. D - F) Cells were treated for 2 hours with DMSO or 10µM SAHA. D) Cell Cycle phase analyzed by flow cytometry. Cells were stained with Guava Cell Cycle Reagent after EtOH fixation. DNA content is shown on a linear scale on the X-axis and cell count on the Y-axis. Cell cycle phases are modeled with FlowJo. E) RPA1$_{Ac\text{-}K163}$ *vs* DNA content in indicated cell treatments showing increased RPA acetylation with SAHA treatment. F) Cells were labelled with EdU for 30 minutes followed by EdU detection with the Click-iT® EdU Flow Cytometry Assay Kit (Molecular Probes). Data is plotted with EdU on the X-axis and RPA1$_{Ac\text{-}K163}$ on the Y-axis. Panels show, from left to right, unstained cells, RPA1$_{Ac\text{-}K163}$ only, EdU only, and dual staining with EdU and RPA1$_{Ac\text{-}K163}$.

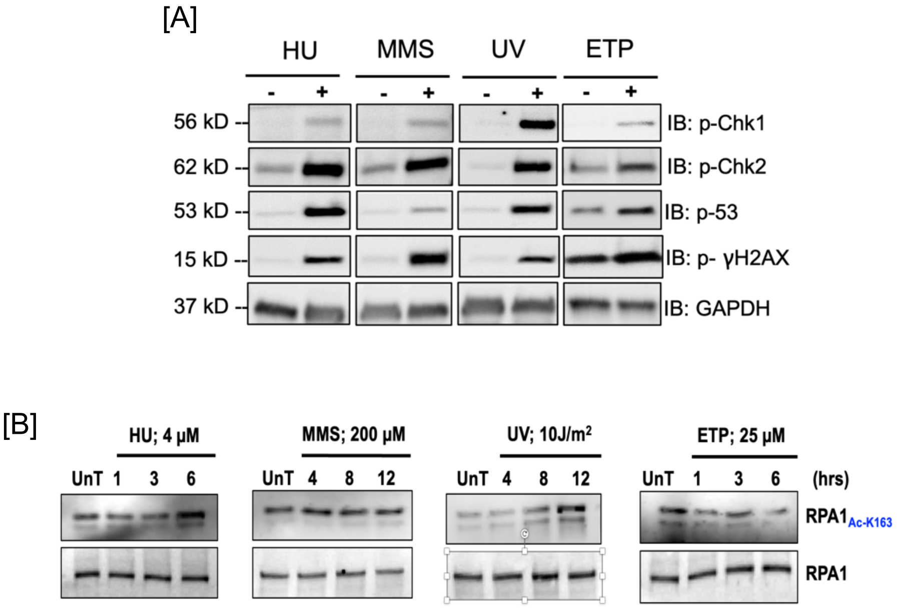

Supplementary Figure 3A: HEK293T cell lysates treated with different DNA damaging agents (as described in Methods) were immunoblotted with (A) antibodies against different DNA damage markers to confirm induction of DNA damage in our experiments. (B) Cell lysates from the different treatments were immunoblotted using the RPA1AC-K163 specific antibody. Graphically plotted in main Figure 3C.

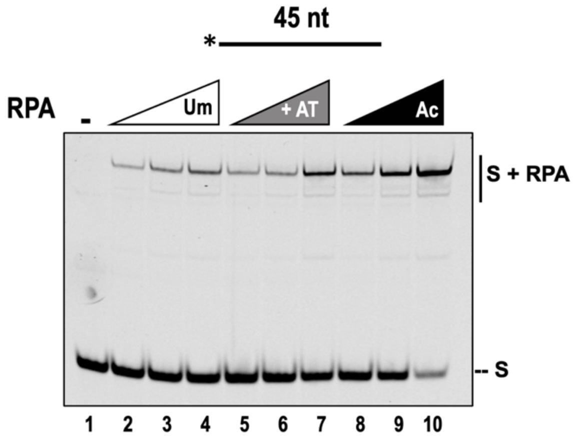

Supplementary Figure 4: RPA acetylation and not the presence of p300 improves ssDNA binding affinity. Five nanomolar of TAMARA labeled 45 nt ssDNA substrate was incubated with increasing concentrations (5, 10, 25 nM) of Um-RPA, AT-RPA (RPA+ p300, in the absence of acetyl CoA) or Ac-RPA, and the reactions were incubated for 10 min at 37°C and reactions were subsequently separated on a 6% polyacrylamide gel. The labeled substrate is depicted above the gel with the asterisk indicating 5' of the TAMARA label. The substrate alone and the complexes containing RPA-bound substrate are indicated beside the gel at the right.

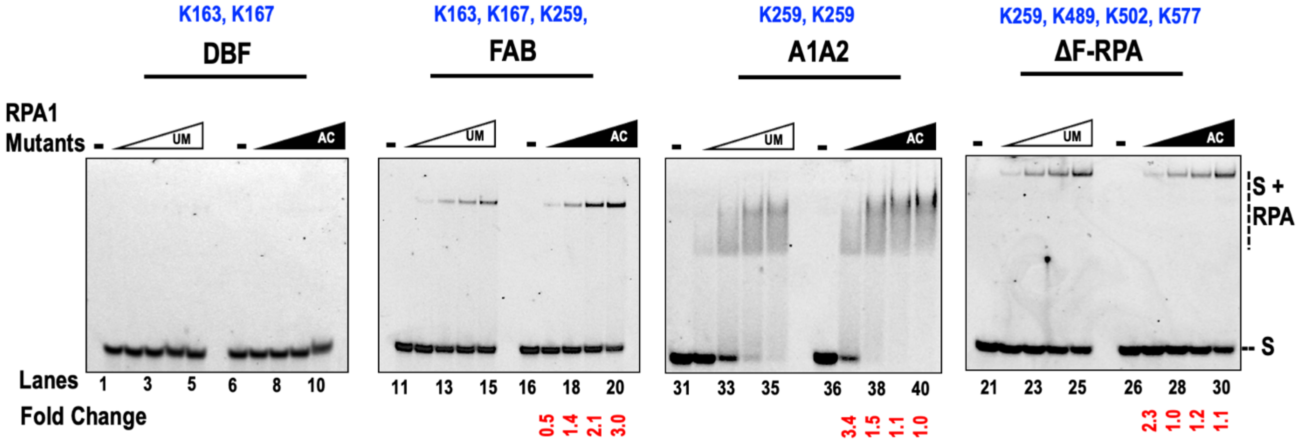

Supplementary Figure 5: Correlating Number of Acetylated Lysine Sites to Increase in Binding Efficiency. Each lysine site acetylated on the mutant form of RPA1 are indicated above every specific mutant RPA. Binding efficiency of unmodified (Um) and acetylated (Ac) forms of mutant RPA was studied using EMSA. Twenty-five nanomolar of 30 nt 5'TAMARA-labeled ssDNA substrate was incubated in with increasing concentrations (25, 50, 100 and 150 nM) of RPA or Ac-RPA, and the reactions were incubated for 10 min at 37°C and separated on a 6% polyacrylamide gel. The substrate alone and the complexes containing RPA-bound substrate are indicated beside the gel at the right. Fold change in the binding of acetylated RPA compared to the unmodified RPA has been denoted below the lane numbers.

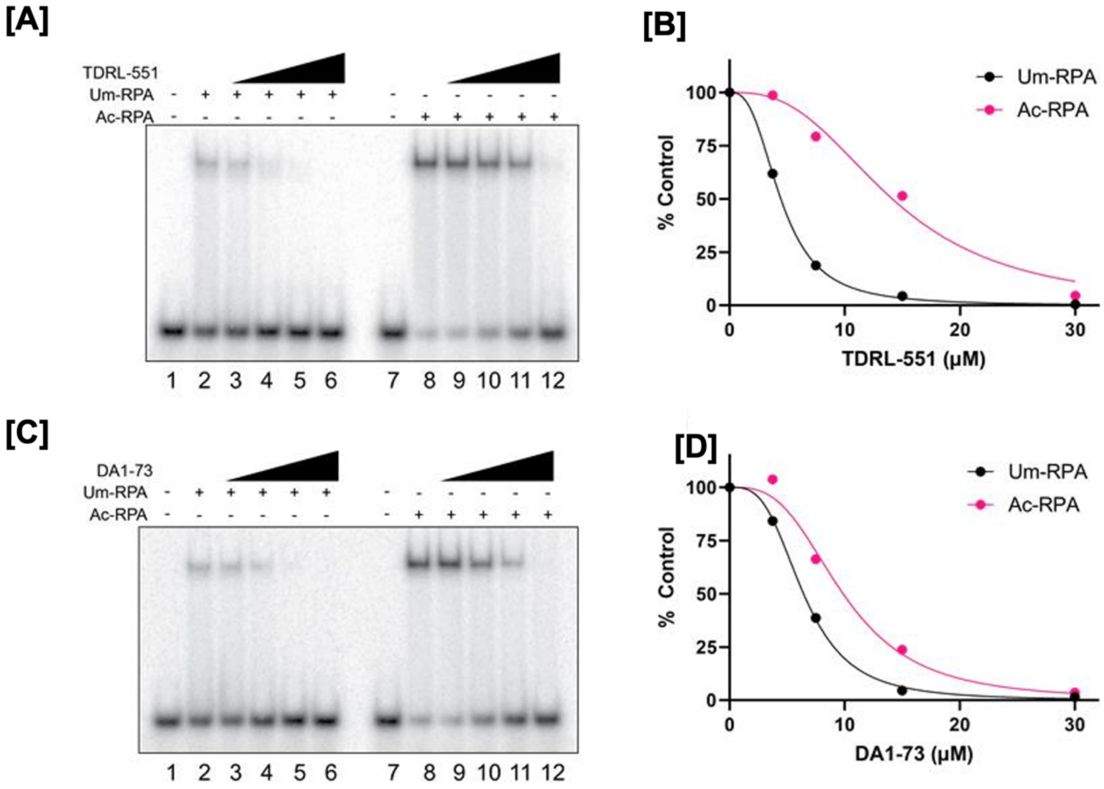

Supplementary Figure 6: **Inhibition of RPA-DNA binding using RPA specific inhibitors.** Representative EMSA titration and TDRL-551 (A-B) or DA1-73 (C-D) inhibition of 12.5nM Um-RPA or Ac-RPA. Data in panels B and D are plotted as average ± SEM from three replicates and fit by non-linear regression.

RPA Inhibitor Assay - EMSAs were performed as previously described with minor modifications (1). Briefly, reactions were performed in 20 mM 4-(2-hydroxyehtyl)-1-piperazineethanesulfonic acid (HEPES) pH 7.8, 50 mM NaCl, 1 mM dithiothreitol (DTT), 0.001% NP-40 with a final volume of 10 µL. TDRL-551 and DA1-73 were suspended in 100% dimethylsulfoxide (DMSO) and diluted fresh immediately before conducting each reaction, and the DMSO concentration in the final reaction mixtures was kept constant at 5%. Purified full-length RPA that was either acetylated (Ac-RPA) or mock treated (Um-RPA) was incubated with the indicated RPAi or vehicle for 30 min at room temperature. Equimolar concentrations of 12.5 nM Um-RPA and Ac-RPA were used. After incubation with the RPAi, 1.25 nM of the $^{[32]}$P-labeled 30-nt ssDNA probe (JJ.30) was added, and reactions were incubated for 5 min at room temperature before products were separated by 6% native polyacrylamide gel electrophoresis. The bound and unbound fractions were quantified by phosphor-imager analysis using ImageQuant software (Molecular Dynamics, CA), and data were fit by non-linear regression using GraphPad Prism.

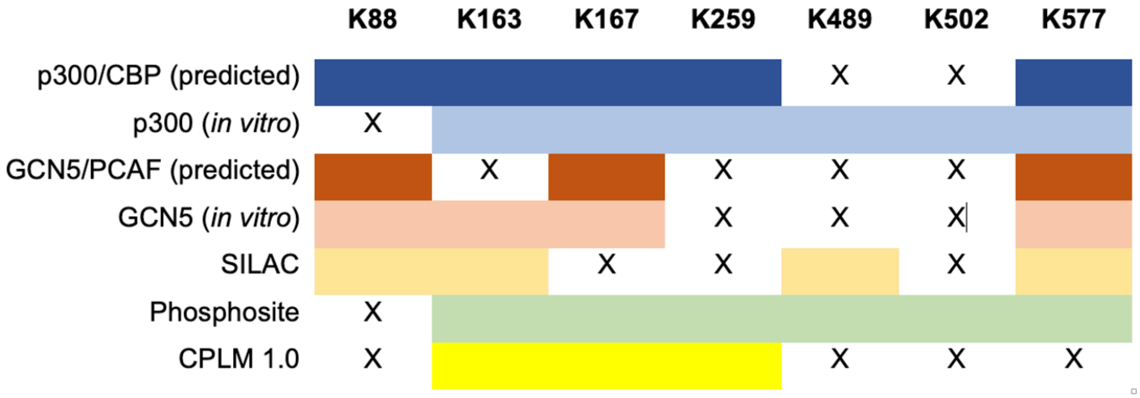

Supplementary Figure 7: Detected and predicted RPA1 lysine acetylation sites.
The GPS-PAIL 2.0 software was used to predict sites of p300 and Gcn5 lysine acetylation activity ((2)). Data from proteomic SILAC studies are presented. Proteomic data deposited in the Phosphosite website ((3)) are presented. Lysine acetylation sites reported in the Compendium of Protein Lysine Modification (CPLM) are presented.

Supplementary Table 1:

All sequences are written in the 5' – 3' direction.

Oligonucleotide	Sequence
LB.20	TTC ACG CCT GTT AGT TAA TT
LB.24	TTC ACG CCT GTT AGT TAA TTC ACT
LB.28	TTC ACG CCT GTT AGT TAA TTC ACT GGC C
LB.30	TTC ACG AGA TTT ACT TAT TTC ACT GCG GCT
JJ.30	GGA GAC CGA AGA GGA AAA GAA GGA GAG AGG
LB.32	TTC ACG CCT GTT AGT TAA TTC ACT GGC CGT AC
LB.37	CAC TGG CCG TCG TTT TAC TTG GAA ACA GAG GTC TCG A
LB.45	TTC ACT ATA ACT ACC TAA TCT TCT GGC CGT ACT GAA CTA CTG ACA
LB.57	TCG AGA CCT CTG TTT CCA AGT AAA ACG ACG GCC AGT GTG CGT AGC GTA CAA TAC CAC
U25	GTCCACCCGACGCCACCTCCTGCCT
D60	AGACGAATTCCGGATACGACGGCCAGTGCCGACCGTGCCAGCCTAAATTTCAATCCACCC
T110	GGGTGGATTGAAATTTAGGCTGGCACGGTCGGCACTGGCCGTCGTATCCGGAATTCGTCTTGGTTGTAGGATCCCAGCACATTGAAGGCAGGAGGTGGCGTCGGGTGGAC
FB110	GGGTGGATTGAAATAGGTCTCGAGGCCTGCTCTATTATGAGCAGGCCTCGAGACCTGGTCTTGGTTGTAGGATCCCAGCACATTGAAGGCAGGAGGTGGCGTCGGGTGGAC
U26	CGCCAGGGTTTTCCCAGTCACGACCA
D43	TTAGTTAATTCACTGGCCGTCGTTTTACAACGACGTGACTGGG
T49	GCCCAGTCACGTCGTTGTAAAACGGGTCGTGACTGGGAAAACCCTGGCG

Oligonucleotides containing TAMRA or IR700 were labelled on the 5' end, while those containing biotin were labelled on the 3' end. Blue font indicates fold-back region.

References:

1. VanderVere-Carozza, P. S., Gavande, N. S., Jalal, S. I., Pollok, K. E., Ekinci, E., Heyza, J. *et al.* (2022) In Vivo Targeting Replication Protein A for Cancer Therapy Front Oncol **12**, 826655 10.3389/fonc.2022.826655
2. Deng, W., Wang, C., Zhang, Y., Xu, Y., Zhang, S., Liu, Z., andXue, Y. (2016) GPS-PAIL: prediction of lysine acetyltransferase-specific modification sites from protein sequences Sci Rep **6**, 39787 10.1038/srep39787
3. Hornbeck, P. V., Kornhauser, J. M., Tkachev, S., Zhang, B., Skrzypek, E., Murray, B. *et al.* (2012) PhosphoSitePlus: a comprehensive resource for investigating the structure and function of experimentally determined post-translational modifications in man and mouse Nucleic Acids Res **40**, D261-270 10.1093/nar/gkr1122
